# Investigating conserved aromatic residues in *ent*-copalyl pyrophosphate synthases required for gibberellin phytohormone biosynthesis

**DOI:** 10.1016/j.phytochem.2025.114635

**Published:** 2025-08-11

**Authors:** Ahmed M.A.A. Raslan, Cody Lemke, Raymond Larsen, Reuben J. Peters

**Affiliations:** Roy J. Carver Department of Biochemistry, Biophysics & Molecular Biology, Iowa State University, Ames, 50011, IA, USA

**Keywords:** Diterpene cyclases, Enzyme evolution, Labdane-related diterpenoids

## Abstract

Terpene synthases and cyclases catalyze carbocation cascade reactions, which have been hypothesized to be directed, in part, by aromatic residues via stabilization of specific intermediates through cation-π interactions towards specific product outcomes. Included in this are class II diterpene cyclases (DTCs), which are particularly widespread due to their role in initiating biosynthesis of gibberellin (GA) phytohormones but also function in production of a vast range of more specialized (labdane-related) diterpenoids. Indeed, the *ent*-copalyl pyrophosphate synthases (CPSs) required for GA biosynthesis are then conserved in all plants, with certain plant-associated bacteria that also produce GA containing potentially distantly related such CPSs as well. Building on the structure determined for the CPS from *Arabidopsis thaliana* (*At*CPS), sequence comparison reveals that all seven aromatic residues in the active sites are conserved, suggesting these may play important roles in the catalyzed reaction. The role of these aromatic residues in directing product outcome was then examined via a series of substitutions for each in two representative examples, one from plants (*At*CPS) and the other bacteria (*Et*CPS from *Erwinia tracheiphila*). Strikingly, substitution with even aliphatic residues had relatively little effect on product outcome, indicating more general structural roles for these aromatic groups. Accordingly, the role of aromatic residues in directing the carbocation cascade reactions catalyzed by at least such CPSs, if not also terpene cyclases and perhaps even synthases, requires additional evidence beyond simple presence in the active site, even when conserved

## 1. Introduction

A role for aromatic residues in directing the carbocation cascade reactions catalyzed by terpene synthases and cyclases via cation-π interactions has long been postulated (Dougherty, 1996), including within DTCs (Pan et al., 2024). However, this hypothesis has not been systematically tested. In part, this is due to the relatively high functional diversity exhibited by these enzymes, coupled to the ability of even single residue changes (not necessarily involving aromatics) to redirect product outcome (Christianson, 2017; Peters, 2025). While conservation of aromatic residues in functionally analogous enzymes might be hypothesized to serve a role in directing the relevant reaction, this is complicated by the parallel convergent evolution of various product outcomes. Specifically, as differing evolutionary paths seem to have often proceeded via distinct position of aromatic residues – i.e., these are not often conserved. In addition, such conservation can also reflect structural requirements, as in the case of the class II cyclases such as DTCs where conserved “QW” (QxxDGSWG) motifs fortify the helical bundle domains of these enzymes (Pan et al., 2024).

DTCs utilize an acid-base mechanism to catalyze bicyclization of the general diterpenoid precursor (*E,E,E*)-geranylgeranyl pyrophosphate (GGPP, **1**), initiating biosynthesis of the labdane-related diterpenoids (Peters, 2010). The catalytic acid is provided by the characteristic DxDD motif, specifically the ‘middle’ aspartic acid (Prisic et al., 2007). By contrast, the catalytic base is not universally conserved, as expected from the various product outcomes mediated by distinct DTCs (Criswell et al., 2012)(see Supplemental Fig. S1).

Fortuitously, the requisite production of GA (or related) hormones in all land plants necessitates the presence of a DTC for production of the relevant *ent*-copalyl pyrophosphate (*ent*-CPP, **2**). It has been shown that the relevant CPSs have been conserved in plants, with some parallels observed to the CPSs from certain plant-associated bacteria that also produce GA, although not those from fungi that similarly produce GA (Lemke et al., 2019). Specifically, building on a crystal structure for *At*CPS (Köksal et al., 2011), a conserved catalytic base dyad consisting of a histidine and asparagine was identified, in large part due to the ability of alanine substitution for either or both to lead to addition of water and production of *ent*-labda-13-en-8β-ol-15-yl pyrophosphate (*ent*-LPP, **3**) (Potter et al., 2014). However, this pair of residues are not required to make **2**, as other DTCs from both plant and bacteria producing this contain alternative residues at these positions, leading to the hypothesis that there may be homology between those involved in GA biosynthesis in both kingdoms (Lemke et al., 2019).

It has been shown that DTCs in plants are derived from the CPSs required for GA biosynthesis (Jia et al., 2022), and the histidine from the ancestral catalytic dyad has often been replaced by other aromatic residues in the derived plant DTCs, which has been demonstrated to impact product outcome (Hansen et al., 2017; Mafu et al., 2015; Pelot et al., 2019; Potter et al., 2016b; Raslan and Peters, 2025; Schulte et al., 2018). However, additional aromatic residues are observed in the active sites of structurally defined CPSs and DTCs more generally (Cowie et al., 2024; Köksal et al., 2011; Ma et al., 2023; Rudolf et al., 2016; Stowell et al., 2022; Tong et al., 2023; Zhou et al., 2012), but their roles have been little explored. Thus, investigation of their conservation and importance for product outcome are described here.

## 2. Results and discussion

**Fig. 1.**
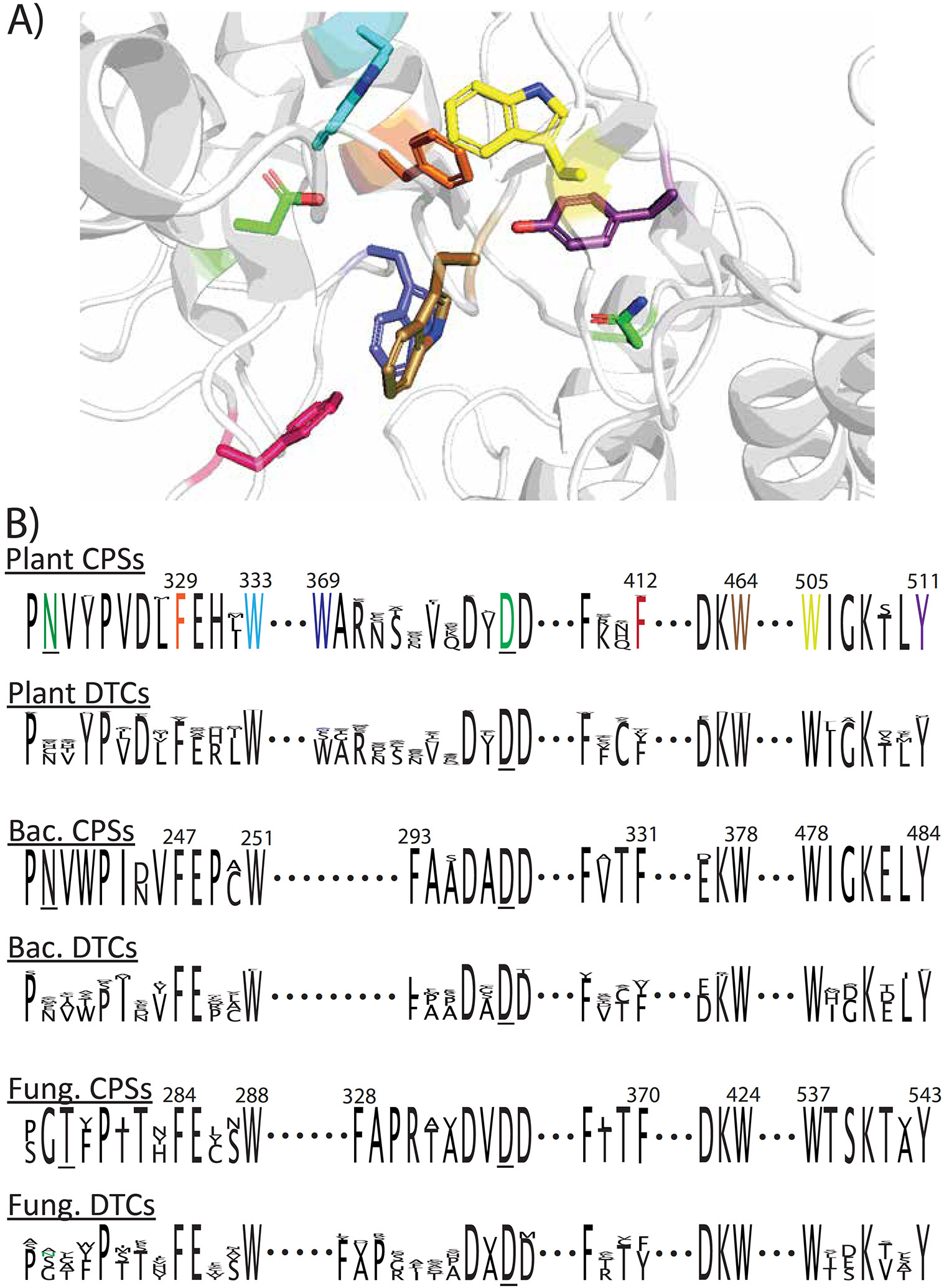
Conserved aromatic residues in active site of *ent*-copalyl pyrophosphate synthases (CPSs) from GA phytohormone biosynthesis. A) Active site of *At*CPS, as defined in part by presence of catalytic acid (D379) and base (N322), shown with carbons colored green, with conserved aromatic residues indicated by distinct coloring of carbons and associated sequence logo (Plant CPSs). B) Sequence logos for associated motifs not only from CPSs but class II diterpene cyclases (DTCs) more generally, as found in plants, bacteria and fungi (as indicated).

Given conservation of the catalytic base dyad in the plant and bacterial CPSs involved in GA biosynthesis within LHS and PNV motifs (Jia et al., 2022), it was hypothesized that these might also contain conserved aromatic residues within their active site serving to stabilize specific carbocation intermediates. For this purpose, such CPSs were defined by not only previously published verification of their production of **2** and the presence of these motifs (see Supplemental Table S1), but in the case of plants also the presence of a specific histidine hypothesized to induce synergistic substrate (**1**) and co-factor (Mg^2+^) inhibition as a biochemical regulatory mechanism for GA biosynthesis (Mann et al., 2010), as observed even in the case of paralogs both producing **2** (Hayashi et al., 2008). The aromatic residues in the active site were first defined by examination of the AtCPS crystal structure (Köksal et al., 2011), with their conservation then determined by sequence alignment. Notably, all seven of the AtCPS active site aromatic residues (Fig. 1A) were essentially completely conserved in plant CPSs (Fig. 1B) – e.g., in AtCPS these are F329, W333, W369, F412, W464, W505 and Y511 – with the only exception being an isoleucine in place of **F**^412^ in one instance (of 41 total). However, the histidine associated with GGPP + Mg^2+^ inhibition in the plant CPSs from GA biosynthesis (H331 in AtCPS) does not form part of the active site, with its side-chain oriented towards the protein surface instead, which suggests an indirect mechanism, potentially mediated by the flanking conserved active site aromatic residues (F329 and W333 in AtCPS).

Strikingly, further extending the sequence similarity between the plant and bacterial CPSs involved in GA biosynthesis, with only one exception, equivalent conserved aromatic residues were found in the 8 verified such bacterial CPSs (e.g., in that from *Erwinia tracheiphila* (*Et*CPS) these are F247, W251, F331, W378, W478 and Y484). Moreover, these occur within larger motifs conserved between these kingdoms (Fig. 1B). The first two are found closely following the second (PNV) catalytic base dyad motif, forming an extended PNV(Y/W)Px(D/N)x**F**Exx**W**^333(251)^ motif, which is more specifically conserved within plants as PNVYPVDL**F**E*H*x**W**^333^ and further contains the regulatory *histidine* noted above, and within bacteria as PNVWPI(D/N)V**F**EPx**W**^251^. The next two aromatic residues are found in separate motifs. The first being Fx(C/T)**F**^412(331)^, more specifically conserved within plant CPSs as FxC**F**^412^ and bacterial CPSs as FxT**F**^331^, while the second is (D/E)K**W**^464(378)^, again more specifically conserved within plant CPSs as DK**W**^464^ and bacterial CPSs as EK**W**^378^. Finally, the last two aromatic residues are found within a broadly conserved **W**IGKxL**Y**^511(484)^ motif. This wider conservation further supports the hypothesized homology between the CPSs from these two kingdoms. Moreover, while the bacterial CPSs lack an exact equivalent to **W**^369^, which is found within a highly conserved **W**AR motif 8 residues upstream of the catalytic acid Dx**D**D motif in plant CPSs, they do contain a conserved phenylalanine (F293 in *Et*CPS) 3 residues upstream instead (Fig. 1B). Note this region forms an extended loop in the *At*CPS crystal structure (Fig. 1A), as well as that of bacterial DTCs such as that from *Streptomyces platensis* producing **2** (Rudolf et al., 2016), leaving their positioning less constrained but still likely differing between the CPSs from each kingdom.

Intriguingly, these aromatic residues also appear to be conserved in the 4 verified fungal CPSs involved in GA biosynthesis (e.g., in the bifunctional CPS-KS from *Gibberella fujikuroi* these are F284, W288, F370, W424, W537 and Y543), despite the second catalytic base dyad motif being missing, albeit the threonine within the corresponding xGT ‘motif’ may serve the same role (Lemke et al., 2019). Although the associated larger motif is less well conserved (Fig. 1B), it still contains these two aromatic residues – i.e., as xGT(F/Y)PxTx**F**Exx**W**^278^. The next two motifs are more completely conserved, with the Fx(C/T)**F** motif present in fungi as FxT**F**^370^ (matching that found in bacteria) and the (D/E)K**W** motif as DK**W**^424^ (matching that found in plants). The final large motif is less well conserved, being present in fungi as **W**TSKTx**Y**^543^ rather than **W**IGKxL**Y**^511(484)^, but nonetheless contains both aromatic residues as well as the ‘middle’ lysine (i.e., this motif is then more broadly conserved as **W**xxKxx**Y**). In addition, these fungal CPSs also contain a conserved Phe (F328 in GfCPS-KS) six residues upstream of the catalytic Dx**D**D motif, intermediate between the spacing observed in plant and bacterial CPSs noted above for **W**^369^ and **F**^293^ (respectively).

To further examine the conservation of these aromatic residues, alignment was carried out with all DTCs (including CPSs) whose activity has been verified by previous publication (see Supplemental Table S1), which revealed some of these residues are not more broadly conserved. In plants the first motif is more broadly conserved as PxxYPxDx**F**x(*H/R*) x**W**^333^ (Fig. 1B), but there is a decrease in conservation of at least **F**^329^, albeit this is still present in 139 of the 153 verified plant DTCs (with tyrosine the most common alternative, found in 10 cases), while **W**^333^ remains present in all but one instance, and the other indicated residues are each found in >94 % [note that *arginine* in place of the *histidine* has been associated with loss of the GGPP + Mg^2+^ inhibition in plant DTCs more generally (Mann et al., 2010)]. Intriguingly, while the rest of the FxC**F**^412^ motif is more highly conserved (>90 %), **F**^412^ is not and is only found in 103 of the 153 plant DTCs, although the most common alternative is tyrosine (32 cases). Similarly, the **W**^69^ eight residues upstream of the DxDD catalytic acid motif also is not more broadly conserved (nor is the associated **W**AR motif), being present in 111 of the 153 plant DTCs, although in all but two cases there is an aromatic residue 7–9 residues upstream. By contrast, the DK**W**^464^ motif is generally conserved, with **W**^464^ present in all but two cases (both tyrosine instead) and the first residue is always (D/E), with arginine in place of the lysine in only one instance. The final motif also is broadly conserved, albeit as **W**xxKxx**Y**^511^ (as in the cross-kingdom comparison of CPSs), with **W**^505^ present in all and **Y**^511^ in 146 of the 153 plant DTCs (with phenylalanine found in 4 cases and histidine in the other 3 instead).

Notably, a similar conservation pattern is observed with the verified bacterial DTCs (Fig. 1B). For example, although the first motif is not more broadly conserved, the aromatic residue containing **F**Exx**W**^251^ submotif is, with **F**^247^ always present and **W**^251^ present in all but one (where it is phenylalanine) of the 25 verified bacterial DTCs (Supplemental Table S1). However, while the rest of the next motif is generally conserved as Fx(T/C)**F**^331^, the active site **F**^331^ is only present in 16 of the 25 (although 8 others contain tyrosine and, in the final case, tryptophan, at this position). Moreover, **F**^293^ is not conserved more generally, with no aromatic residue closely upstream of the DxDD motif in other verified bacterial DTCs. But much as found in plants, the remaining motifs and residues are broadly conserved, including the presence in all verified bacterial DTCs of **W**^378^ from the (D/E)K**W**^378^ motif and **W**^478^ from the **W**xxKxx**Y**^484^ motif, with **Y**^484^ present in all but one instance (where this is phenylalanine in any case). Notably, an even more similar conservation pattern was observed with the 15 verified fungal DTCs (Fig. 1B). Briefly, the aromatic residues within the **F**Exx**W**^278^, DK**W**^424^, and **W**xxKxx**Y**^543^ motifs are completely conserved, while decreased conservation is observed with that from the Fx(T/C)**F**^370^ motif, with only 5 containing **F**^370^ (although the rest contain tyrosine), but **F**^328^ is more generally present in of the verified fungal DTCs (13/15 cases, with the only two exemptions being a tyrosine or leucine), albeit sometimes situated seven instead of six residues upstream of the catalytic acid Dx**D**D motif.

To investigate the role of the seven active site aromatic residues conserved in plant and/or bacterial CPSs if not also DTCs more broadly, in both *At*CPS and *Et*CPS each of these was substituted with up to five alternative amino acids – i.e., alanine, leucine, histidine, phenylalanine and tyrosine – excluding cases where the original residue was already phenylalanine or tyrosine. These were chosen for their expected range of effects, from almost complete removal to just loss of aromaticity as well introduction or removal of functional groups. Tryptophan was excluded due to the significant increase in steric bulk, which was expected to occlude the active site and prevent substrate binding. The impact of these substitutions on product outcome was then analyzed by expression of the resulting variants in *Escherichia coli* also engineered to produce GGPP (**1**). The substrate and any products were detected by extraction of the primary alcohol derivatives stemming from dephosphorylation by endogenous phosphatases and are indicated by prime notation (e.g., geranylgeraniol, **1’**), as observed via GC-MS analysis.

**Fig. 2.**
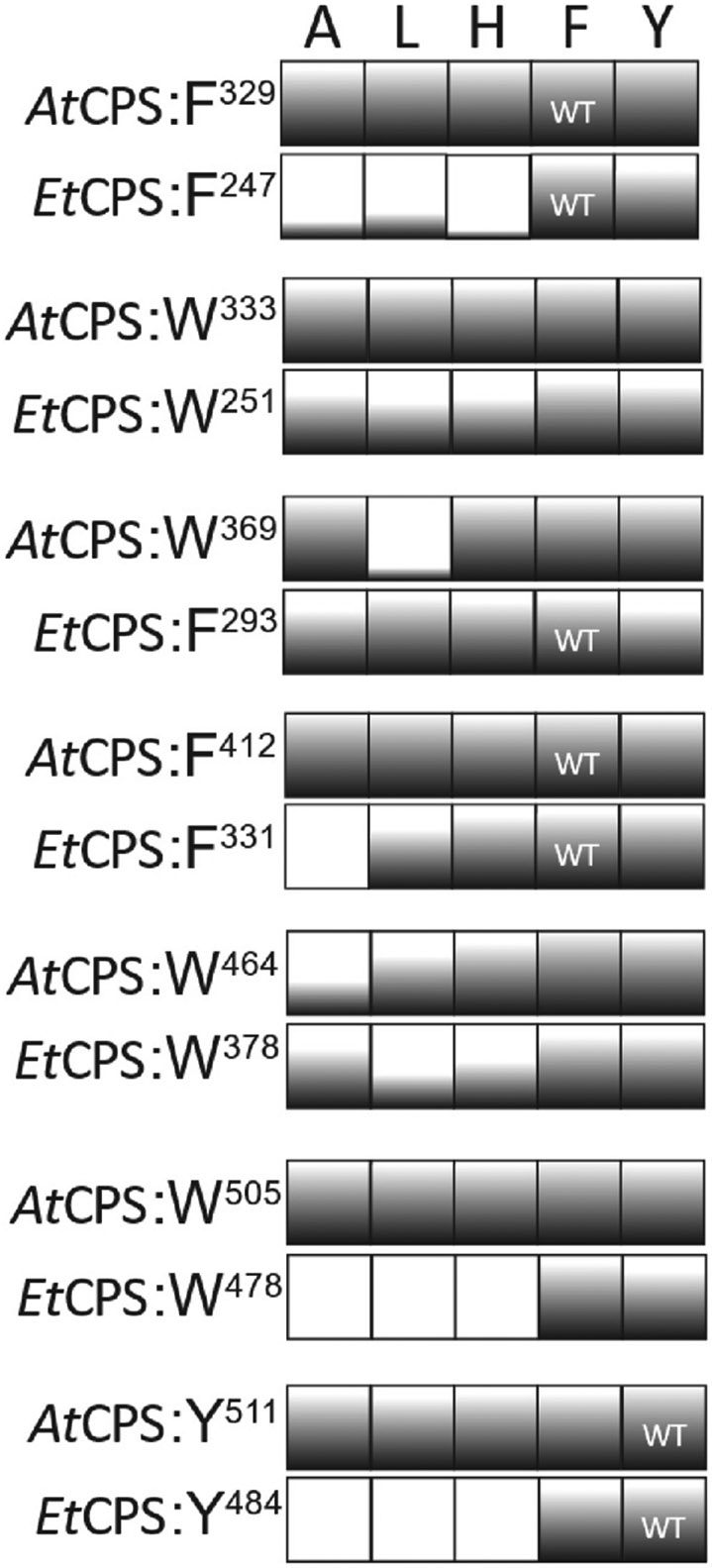
Effect of substitutions on turnover. Calculated from peak areas in total ion count GC-MS chromatograms as percentage – i.e., product peak area(s) ÷ total (substrate and products) peak areas – and rounded to the nearest multiple of 5 % for depiction here.

Surprisingly, most of these substitutions had little to no impact on product outcome, as indicated by continued selective conversion of **1** into *ent*-CPP (**2**) – i.e., observation of **2’** (see Supplemental Fig. S2 for effect of all variants). However, in many cases this is accompanied by loss of relative activity, as indicated by incomplete or complete lack of turnover – i.e., observation of the substrate GGPP as **1’**. In general, such loss of activity was inversely correlated with residue size (e.g., alanine was most deleterious) and more prevalent with equivalent variants of the bacterial *Et*CPS relative to the plant *At*CPS (Fig. 2). There are only two exceptions. The first is leucine substitution for **W**^369^, which is from the **W**AR motif specific to plant CPSs and whose equivalence to **F**^293^ is inexact given their distinct spacing relative to the catalytic acid DxDD motif (Fig. 1). The other is alanine substitution for **W**^464^. Notably, substitution of leucine and, to a lesser extent, histidine also for **W**^464^ are the only other variants with significantly reduced activity in *At*CPS. **W**^464^ is from the DK**W** motif, which sits within a loop thought to open outward to permit substrate entry and then fold inward, with the lysine residue binding to the pyrophosphate moiety in a "lid-closing" mechanism (Koksal et al., 2011).

**Fig. 3.**
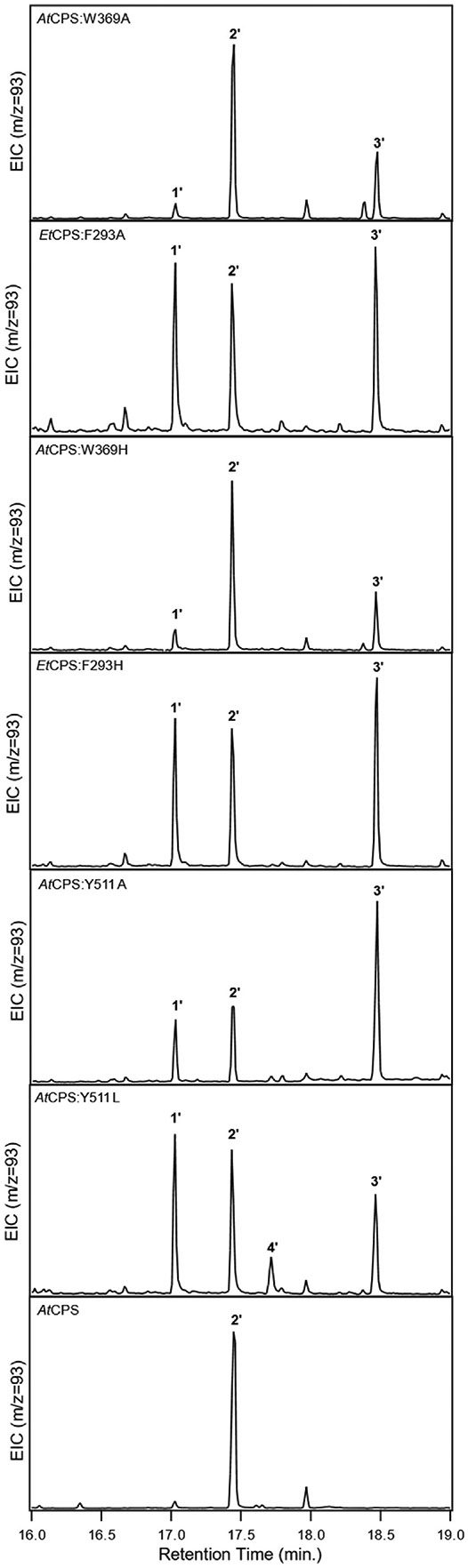
Effect of substitutions for conserved active site aromatic residues in representative CPSs from either plants (*At*CPS) or bacteria (*Et*CPS), with wild-type *At*CPS for comparison. Selected ion chromatograms from analysis via gas chromatography with mass spectral detection for those variants producing *ent*-labda-13-en-8β-ol-15-yl pyrophosphate (**3**) [and in one case also *ent*-labda-7,13-dienyl pyrophosphate (**4**)], in addition to *ent*-copalyl pyrophosphate (**2**), with decreased activity indicated by presence of the substrate (*E,E,E*)-geranylgeranyl pyrophosphate (**1**), all observed as dephosphorylated derivatives as indicated by the prime notation (i.e., **1′**, **2′**, **3′** and **4′**). Note that selection of *m/z* = 93 inflates relative peak area for the substrate (**1′**) versus the products (i.e., **2′**, **3′** and **4′**).

Nonetheless, certain substitutions were sufficient to alter product outcome (Fig. 3). For example, substitution of alanine or histidine for both **W**^369^ in *At*CPS and **F**^293^ in *Et*CPS, despite their inexact equivalences noted above, led to significant production of the hydroxylated derivative *ent*-LPP (**3**; observed as **3′**) alongside **2**, resulting from addition of water to the initially formed *ent*-labda-13-en-15-PP-8-yl carbocation intermediate prior to terminating deprotonation (Fig. S1). Similarly, substitution of alanine or leucine for **Y**^511^ in *At*CPS, which is positioned near **W**^369^ (Fig. 1A), also led to some production of **3′**, with *At*CPS:Y511L further producing small amounts of *ent*-labda-7,13-dienyl pyrophosphate (**4**, observed as **4’**) as well. Consistent with these observations, substitution of alanine for the equivalent tryptophan (i.e., *At*CPS **W**^369^) in another plant DTC has been previously reported to lead to generation of **3** (Pelot et al., 2017), while substitution of glycine for the equivalent phenylalanine (i.e., *At*CPS **Y**^511^) in two other plant DTCs also altered product outcome, with substitution of serine for the histidine equivalent in another further exerting a similar effect (Pelot et al., 2018). This suggests the hypothesis that these substitutions simply allow sufficient room for water to bind proximal to the carbocation in the initial bicyclic intermediate *ent*-labda-13-en-15-PP-8-yl^+^, enabling addition and/or use as an alternative catalytic base, which is supported by the proximity of **W**^369^ and **Y**^511^ to the catalytic base dyad in *At*CPS (Fig. 1A). However, while the presence of histidine in place of the *At*CPS **Y**^511^ has been shown to be important for product outcome in the rice *syn*-CPP synthase (Potter et al., 2016a), this substitution (i.e., Y511H) had no significant effect in *At*CPS (Supplemental Fig. S2). Moreover, although the equivalent tyrosine to the *Et*CPS **Y**^484^ serves as the catalytic base in the halima-5,13-dienyl pyrophosphate synthase from *Mycobacterium tuberculosis* (Lemke et al., 2022), none of the substitutions for this in *Et*CPS altered product outcome, although several lost all activity (Fig. 2).

## 3. Conclusions

Given the conservation of the active site aromatic residues targeted here, it seems surprising how little effect most substitutions had on product outcome. This may reflect the highly exothermic (>30 kcal mol^−1^) nature of the catalyzed bi-cyclization, which then may not need to rely on cation-π stabilization of intermediates following initiation of the reaction – i.e., beyond that exerted by the catalytic base dyad (Potter et al., 2016b). Although some alteration was observed, this was generally limited to the addition of water and, hence, production of **3**, presumably as result of simply opening space for a water to bind in a suitable position. Thus, while more significant effects might be observed with substitutions for more than one of these conserved active site aromatic residues, the results reported here with representative CPSs from plant and bacterial GA biosynthesis do not support a role for these in directing the catalyzed carbocation cascade reaction (at least individually), instead indicating they play more general roles, either structural or in the initial concerted bi-cyclization – i.e., via cation-π stabilization of the transition state for the initiating protonation, the absence of which might underlie some of the loss of activity observed here but does not direct product outcome per se in any case. Altogether, this study emphasizes the importance of more rigorous investigation of the oft hypothesized role for aromatic residues in directing product outcome in the carbocation cascade reactions catalyzed by terpene synthases and cyclases.

## 4. Methods

### 4.1. General

All reagents were purchased from Fisher Scientific unless otherwise mentioned.

### 4.2. Recombinant constructs and site-directed mutagenesis

The Invitrogen Gateway System was utilized to construct all the vectors used here. The wild-type constructs were those previously described (Nagel and Peters, 2017; Prisic and Peters, 2007). Mutagenesis was performed with pENTR/SD/D-TOPO constructs via whole-plasmid PCR with overlapping primers. The resulting variants were confirmed by whole-gene sequencing carried out by the DNA facility at Iowa State University. Each verified mutant was then transferred into pDEST14 via an LR clonase reaction.

### 4.3. Metabolic engineering

CPS activity was analyzed via a previously described metabolic engineering system (Cyr et al., 2007). Briefly, each construct was introduced individually into the C41 OverExpress strain of *E. coli* (Lucigen) in combination with the pGG vector, which expresses a GGPP synthase. The resulting recombinant strains were grown in a 5 mL pre-culture (NZY media and the appropriate antibiotics) at 180 RPM and 37 °C for 16 h. This was then added to 45 mL of TB media, containing 100 mM of phosphate buffer (pH 7.0) and antibiotics, in 250 mL Erlenmeyer flasks, and grown (180 RPM and 37 °C) until an OD_600_ of 0.6–0.8 was reached. The temperature was then lowered to 16 °C for 1 h, and the cultures induced with IPTG at a final concentration of 1 mM. The cultures were grown for an additional 72 h (180 RPM and 16 °C). Products were extracted by shaking with 50 mL hexanes for 20 min at 180 RPM and 37 °C. After initial removal of the organic (hexanes) layer, 0.2 mL of ethanol was added to the remaining emulsion to facilitate further separation, which was repeated a second time if necessary (i.e., to obtain >49 mL of organic extract). The hexane extract was divided equally between 3 glass test tubes, dried under a stream of N2 gas, resuspended in 600 μL of hexane, and transferred to a vial for analysis via gas chromatography with mass spectral detection (GC-MS).

### 4.4. GC-MS analysis

The GC-MS equipment and parameters used for all analyses were as previously described (Raslan and Peters, 2025). Briefly, using a 8890 GC System equipped with a 5977B mass spectrometer (Agilent) operating in 70 eV electron ionization mode and using an HP-5MS column at a flow rate of 1.1 mL/min of helium. Samples were injected in splitless mode at a temperature of 250 °C using a 7650A automatic liquid sampler. The oven temperature was maintained at 50 °C for 3 min, followed by a ramp of 15 °C/min to 300 °C, which was maintained for an additional 3 min. Data from the mass spectrometer were recorded for mass-to-charge (m/z) ratios between 90 and 600, starting 13 min after sample injection until the end of the run.

### 4.5. Bioinformatic analyses

The DTC origin, products, accession numbers and corresponding references for their biochemical characterization can be found in Supplemental Table S1. These were aligned using the Create Alignment (open gap cost = 10 and gap extension cost = 1) tool in CLC Main Workbench, version 25.0.1 (QIAGEN), and the relevant sequence logos extracted for presentation here.

## Supplementary Material

SI

Supplementary data to this article can be found online at https://doi.org/10.1016/j.phytochem.2025.114635.

## Data Availability

Data will be made available on request.
