# Supplementary material for: Investigating conserved aromatic residues in ent-copalyl pyrophosphate synthases required for gibberellin phytohormone biosynthesis
Source: Phytochemistry. Author manuscript; Available in PMC 2025 Sep 5. (PMC12410513; doi:10.1016/j.phytochem.2025.114635)
Supplement: SI [file NIHMS2104951-supplement-SI.pdf]

Supporting information for:

Investigating conserved aromatic residues in *ent*-copalyl pyrophosphate synthases required for gibberellin phytohormone biosynthesis

Ahmed M.A.A. Raslan, Cody Lemke[±], Raymond Larsen and Reuben J. Peters*

Roy J. Carver Department of Biochemistry, Biophysics & Molecular Biology, Iowa State University, Ames, IA 50011, USA

Table of Contents

Page 1 Figure S1: Basic DTC mechanism

Page 2-8 Table S1: Verified DTCs

Page 9-12 Figure S2: GC-MS chromatograms for all described mutants

Page 13-22 References

Figure S1: Basic mechanism for DTC catalyzed reactions and derived products.

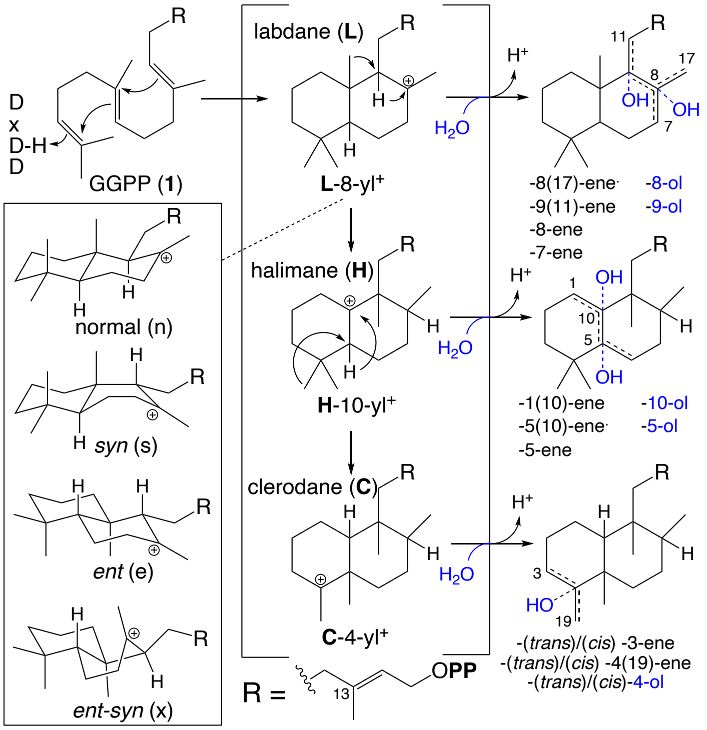

Table S1: Verified class II diterpene cyclases

Name	Product(see scheme)	GA/2°	Kingdom[1]	Species	Accession	Ref.
**AtCPS**	*ent*-**L**-8(17)-ene	GA	Plant-c	*A. thaliana*	NP_192187	(Sun and Kamiya, 1994)
**EtCPS**	*ent*-**L**-8(17)-ene	GA	Bact.	*E. tracheiphila*	WP_020322919	(Nagel and Peters, 2017)
JsCPSKS	*ent*-**L**-8(17)-ene	GA	Plant-c	*Jungermannia subulata*	BAJ39816	(Kawaide et al., 2011)
PcCPS	*ent*-**L**-8(17)-ene	GA	Plant-c	*Phaeoceros carolinianus*	UPQ49770	(Jia et al., 2022)
LcCPS	*ent*-**L**-8(17)-ene	GA	Plant-c	*Lunularia cruciata*	UPQ49771	(Jia et al., 2022)
MpCPS1	*ent*-**L**-8(17)-ene	GA	Plant-c	*Marchantia paleacea*	UPQ49772	(Jia et al., 2022)
MpCPS	*ent*-**L**-8(17)-ene	GA	Plant-c	*Marchantia polymorpha*	APP91795	(Kumar et al., 2016)
LjCPSKS	*ent*-**L**-8(17)-ene	GA	Plant-c	*Leucodon julaceus*	UPQ49778	(Jia et al., 2022)
SwCPS	*ent*-**L**-8(17)-ene	GA	Plant-c	*Selaginella wallacei*	UPQ49773	(Jia et al., 2022)
VaCPS	*ent*-**L**-8(17)-ene	GA	Plant-c	*Vittaria appalachiana*	UPQ49774	(Jia et al., 2022)
SmoCPS1	*ent*-**L**-8(17)-ene	GA	Plant-c	*Selaginella moellendorffii*	AFR34002	(Li et al., 2012a)
SmoCPS2	*ent*-**L**-8(17)-ene	GA	Plant-c	*Selaginella moellendorffii*	AFR34003	(Li et al., 2012a)
PpCPSKS	*ent*-**L**-8(17)-ene	GA/(*R*)[2]	Plant-c	*Physcomitrella patens*	XP_024380398	(Hayashi et al., 2006)
PlCPSKS	*ent*-**L**-8(17)-ene	GA/(*N*)	Plant-c	*Pallavicinia lyellii*	UPQ49777	(Jia et al., 2022)
AfCPSKS	*ent*-**L**-8(17)-ene	GA/(*R*)	Plant-c	*Azolla filiculoides*	UPQ49779	(Jia et al., 2022)
PcSS	*ent*-**L**-8(17)-ene	2°	Plant-c	*Phaeoceros carolinianus*	UPQ49783	(Jia et al., 2022)
OpKOS	*ent*-**C**-3-ene	2°	Plant-c	*Odontoschisma prostratum*	UPQ49775	(Jia et al., 2022)
MpDTPS6	*cis*-*e*/*n*-**C**-3-ene	2°	Plant-c	*Marchantia polymorpha*	UPQ49784	(Jia et al., 2022)
MpDTPS2	*e*/*n*-**C**-3-ene	2°	Plant-c	*Marchantia polymorpha*	UPQ49776	(Jia et al., 2022)
MpDTPS7	*syn*-**C**-3-ene	2°	Plant-c	*Marchantia polymorpha*	UPQ49786	(Jia et al., 2022)
OlIAS	*ent*-**L**-8β-ol	2°	Plant-c	*Orthotrichum lyellii*	UPQ49787	(Jia et al., 2022)
VaIPS	*ent*-**L**-8(17)-ene	2°	Plant-c	*Vittaria appalachiana*	UPQ49788	(Jia et al., 2022)
SmCPSKSL1	*e*/*n*-**L**-7-ene	2°	Plant-h	*Selaginella moellendorffii*	AEK75338	(Mafu et al., 2011)
SmCPSMS	*e*/*n*-**L**-8(17)-ene	2°	Plant-h	*Selaginella moellendorffii*	BAL41682	(Sugai et al., 2011)
CpCPSPS	*syn*-**L**-8(17)-ene	2°	Plant-h	*Calohypnum plumiforme*	BAV01232	(Okada et al., 2016)
MpDTPS5	*syn*-**C**-3-ene	2°	Plant-h	*Marchantia polymorpha*	UPQ49785	(Jia et al., 2022)
OsCPS	*e*/*n*-**C**-8-ene	2°	Plant-h	*Osmunda sp.*	UPQ49789	(Jia et al., 2022)

HsLS	*n*-**L**-8(17)-ene	2°	Plant-h	*Huperzia squarrosa*	UPQ49780	(Jia et al., 2022)
PdLS	*n*-**L**-8(17)-ene	2°	Plant-h	*Phylloglossum drummondii*	UPQ49781	(Jia et al., 2022)
OjMS	*e*/*n*-**L**-8(17)-ene	2°	Plant-h	*Osmunda japonica*	UPQ49782	(Jia et al., 2022)
TcCPS3	*ent*-**L**-8(17)-ene	GA	Plant-c	*Taiwania cryptomerioides*	AOG18237	(Ma et al., 2019)
PgCPS	*ent*-**L**-8(17)-ene	GA/(*R*)	Plant-c	*Picea glauca*	ADB55707	(Keeling et al., 2010)
PsCPS	*ent*-**L**-8(17)-ene	GA/(*R*)	Plant-c	*Picea sitchensis*	ADB55709	(Keeling et al., 2010)
AgAS	*n*-**L**-8(17)-ene	2°	Plant-d3	*Abies grandis*	AAB05407	(Vogel et al., 1996)
AbAS	*n*-**L**-8(17)-ene	2°	Plant-d3	*Abies balsamea*	AEL99950	(Zerbe et al., 2012)
AbPS	*n*-**L**-8(17)-ene	2°	Plant-d3	*Abies balsamea*	AEL99951	(Zerbe et al., 2012)
AbCS	*n*-**L**-8β-ol	2°	Plant-d3	*Abies balsamea*	AEL99953	(Zerbe et al., 2012)
GbLS	*n*-**L**-8(17)-ene	2°	Plant-d3	*Ginkgo biloba*	AAL09965	(Schepmann et al., 2001)
PaAS	*n*-**L**-8(17)-ene	2°	Plant-d3	*Picea abies*	AAS47691	(Martin et al., 2004)
PaPS	*n*-**L**-8(17)-ene	2°	Plant-d3	*Picea abies*	AAS47690	(Martin et al., 2004)
PtAS	*n*-**L**-8(17)-ene	2°	Plant-d3	*Pinus taeda*	AAX0743	(Ro and Bohlmann, 2006)
PbAS	*n*-**L**-8(17)-ene	2°	Plant-d3	*Pinus banksiana*	AFU73864	(Hall et al., 2013)
PcAS1	*n*-**L**-8(17)-ene	2°	Plant-d3	*Pinus contorta*	AFU73862	(Hall et al., 2013)
PcAS2	*n*-**L**-8(17)-ene	2°	Plant-d3	*Pinus contorta*	AFU73863	(Hall et al., 2013)
PsAS	*n*-**L**-8(17)-ene	2°	Plant-d3	*Picea sitchensis*	F2XFB2	(Keeling et al., 2011)
PsPS	*n*-**L**-8(17)-ene	2°	Plant-d3	*Picea sitchensis*	F2XF92	(Keeling et al., 2011)
PxAS	*n*-**L**-8(17)-ene	2°	Plant-d3	*Pseudolarix amabilis*	AGN70885	(Zerbe et al., 2013)
TcLPS2	*n*-**L**-8β-ol	2°	Plant-d3	*Taiwania cryptomerioides*	AOG18229	(Ma et al., 2019)
TcCPS4	*n*-**L**-8(17)-ene	2°	Plant-d3	*Taiwania cryptomerioides*	AFE61356	(Ma et al., 2019)
TpCPS	*n*-**L**-8(17)-ene	2°	Plant-d3	*Thuja plicata*	QND75953	(Tasnim et al., 2020)
CfCPS1	*n*-**L**-8(17)-ene	2°	Plant-d3	*Chamaecyparis formosensis*	QWV53997	(Ma et al., 2021)
CtCPS	*n*-**L**-8(17)-ene	2°	Plant-d3	*Chamaecyparis taiwanensis*	WRK57351	(Wu et al., 2024)
CoCPS	*n*-**L**-8(17)-ene	2°	Plant-d3	*Chamaecyparis obtusa*	WRK57353	(Wu et al., 2024)
PsCPS	*ent*-**L**-8(17)-ene	GA	Plant-c	*Pisum sativum*	NP_001414643	(Ait-Ali et al., 1997)
CmCPS1	*ent*-**L**-8(17)-ene	GA	Plant-c	*Cucurbita maxima*	XP_022967380	(Smith et al., 1998)
CmCPS2	*ent*-**L**-8(17)-ene	GA	Plant-c	*Cucurbita maxima*	XP_022995588	(Smith et al., 1998)

SrCPS	*ent*-**L**-8(17)-ene	GA	Plant-c	*Stevia rebaudiana*	AAB87091	(Richman et al., 1999)
LeCPS	*ent*-**L**-8(17)-ene	GA	Plant-c	*Lycopersicon esculentum*	BAA84918	(Rebers et al., 1999)
LsCPS	*ent*-**L**-8(17)-ene	GA	Plant-c	*Lactuca sativa*	BAB12440	(Sawada et al., 2008)
PtCPS1	*ent*-**L**-8(17)-ene	GA	Plant-c	*Populus trichocarpa*	ALM22923	(Irmisch et al., 2015)
SduCPS	*ent*-**L**-8(17)-ene	GA	Plant-c	*Scoparia dulcis*	BAD91286	(Nakagiri et al., 2005)
ApCPS2	*ent*-**L**-8(17)-ene	GA	Plant-c	*Andrographis paniculata*	AFH53508	(Misra et al., 2015)
EpCPS	*ent*-**L**-8(17)-ene	GA	Plant-c	*Euphorbia peplus*	AGN70883	(Zerbe et al., 2013)
GhCPS	*ent*-**L**-8(17)-ene	GA	Plant-c	*Gossypium hirsutum*	NP_001314144	(Wang et al., 2014)
SmCPS5	*ent*-**L**-8(17)-ene	GA	Plant-c	*Salvia miltiorrhiza*	ALX18648	(Su et al., 2016)
TwCPS	*ent*-**L**-8(17)-ene	GA	Plant-c	*Tripterygium*	ANO43020	(Hansen et al., 2017)
TrCPS1	*ent*-**L**-8(17)-ene	GA	Plant-c	*Tripterygium regelii*	ASP43411	(Inabuy et al., 2017)
TwCPSv2	*ent*-**L**-8(17)-ene	GA	Plant-c	*Tripterygium wilfordii*	AKM28414	(Su et al., 2018)
IrCPS	*ent*-**L**-8(17)-ene	GA	Plant-c	*Isodon rubescens*	ARO38143	(Pelot et al., 2017a)
IrCPS4	*ent*-**L**-8(17)-ene	GA	Plant-c	*Isodon rubescens*	APJ36374	(Jin et al., 2017)
IrCPS5	*ent*-**L**-8(17)-ene	GA	Plant-c	*Isodon rubescens*	APJ36375	(Jin et al., 2017)
IeCPS1	*ent*-**L**-8(17)-ene	GA	Plant-c	*Isodon eriocalyx*	AEP03177	(Li et al., 2012b)
IlCPS6	*ent*-**L**-8(17)-ene	GA	Plant-c	*Isodon lophanthoides*	QXT24224	(Yang et al., 2021)
CaCPS	*ent*-**L**-8(17)-ene	GA	Plant-c	*Chiococca alba*	QIA61386	(Lau et al., 2020)
CamCPS1	*ent*-**L**-8(17)-ene	GA	Plant-c	*Callicarpa americana*	QMW69081	(Hamilton et al., 2020)
CamCPS3	*ent*-**L**-8(17)-ene	GA	Plant-c	*Callicarpa americana*	QMW69080	(Hamilton et al., 2020)
SsLPS	*n*-**L**-8β-ol	2°	Plant-c	*Salvia sclarea*	AET21247	(Schalk et al., 2012)
SsLPSv2	*n*-**L**-8β-ol	2°	Plant-c	*Salvia sclarea*	AFU61897	(Caniard et al., 2012)
GrLPS	*n*-**L**-8β-ol	2°	Plant-c	*Grindelia robusta*	AGN70887	(Zerbe et al., 2013)
GrCPS2	*n*-**L**-7-ene	2°	Plant-c	*Grindelia robusta*	AKP96361	(Zerbe et al., 2015)
SmCPS1	*n*-**L**-8(17)-ene	2°	Plant-c	*Salvia miltiorrhiza*	ABV57835	(Gao et al., 2009)
SmCPS2	*n*-**L**-8(17)-ene	2°	Plant-c	*Salvia miltiorrhiza*	AHJ59322	(Cui et al., 2015)
SmLPS	*ent*-**L**-8β-ol	2°	Plant-c	*Salvia miltiorrhiza*	AKN91186	(Cui et al., 2015)
SfCPS	*n*-**L**-8(17)-ene	2°	Plant-c	*Salvia fruticosa*	AJQ30184	(Bozic et al., 2015)
SrCPS1	*n*-**L**-8(17)-ene	2°	Plant-c	*Salvia rosmarinus*	AHL67261	(Bozic et al., 2015)
MvCPS	*n*-**L**-8(17)-ene	2°	Plant-c	*Marrubium vulgare*	AIE77092	(Zerbe et al., 2014)
MvPPS	*syn*-**L**-9α-ol	2°	Plant-c	*Marrubium vulgare*	AIE77090	(Zerbe et al., 2014)

CfCPS1	*n*-**L**-8(17)-ene	2°	Plant-c	*Coleus forskohlii*	AHW04046	(Pateraki et al., 2014)
CfCPS2	*n*-**L**-8(17)-ene	2°	Plant-c	*Coleus forskohlii*	AZB50379	(Johnson et al., 2019)
CfLPS	*n*-**L**-8β-ol	2°	Plant-c	*Coleus forskohlii*	AHW04047	(Pateraki et al., 2014)
SrLPS	*n*-**L**-8β-ol	2°	Plant-c	*Stevia rebaudiana*	ALJ30096	(Kim et al., 2015)
SdCPS1	*ent*-**L**-8(17)-ene	2°	Plant-c	*Salvia divinorum*	APH81399	(Pelot et al., 2017b)
SdKPS	*ent*-**C**-3-ene	2°	Plant-c	*Salvia divinorum*	APH81400	(Pelot et al., 2017b)
SdKSPv2	*ent*-**C**-3-ene	2°	Plant-c	*Salvia divinorum*	AOZ15895	(Chen et al., 2017)
VaCPS3	*syn*-**L**-8(17)-ene	2°	Plant-c	*Vitex agnus-castus*	AUT77122	(Heskes et al., 2018)
VaPPS	*syn*-**L**-9α-ol	2°	Plant-c	*Vitex agnus-castus*	AUT77120	(Heskes et al., 2018)
VaKPS	*n*-**C**-3-ene	2°	Plant-c	*Vitex agnus-castus*	AUT77124	(Heskes et al., 2018)
TwKPS2	*ent*-**C**-3-ene	2°	Plant-c	*Tripterygium wilfordii*	ALE19956	(Andersen-Ranberg et al., 2016)
TwKPS1	*ent*-**C**-3-ene	2°	Plant-c	*Tripterygium wilfordii*	ANO43021	(Hansen et al., 2017)
TwLPS	*n*-**L**-8β-ol	2°	Plant-c	*Tripterygium wilfordii*	ALE19957	(Andersen-Ranberg et al., 2016)
TwCPS1	*n*-**L**-8(17)-ene	2°	Plant-c	*Tripterygium wilfordii*	ANO43022	(Hansen et al., 2017)
TwCPS2	*n*-**L**-8(17)-ene	2°	Plant-c	*Tripterygium wilfordii*	ALE19955	(Andersen-Ranberg et al., 2016)
TwCPS1v2	*n*-**L**-8(17)-ene	2°	Plant-c	*Tripterygium wilfordii*	AQW38541	(Su et al., 2018)
TwCPS2v2	*n*-**L**-8(17)-ene	2°	Plant-c	*Tripterygium wilfordii*	AKM28412	(Su et al., 2018)
TrCPS2	*n*-**L**-8(17)-ene	2°	Plant-c	*Tripterygium regelii*	ASP43410	(Inabuy et al., 2017)
IeCPS2	*ent*-**L**-8(17)-ene	2°	Plant-c	*Isodon eriocalyx*	AEP03175	(Li et al., 2012b)
IrCPS1	*n*-**L**-8(17)-ene	2°	Plant-c	*Isodon rubescens*	ARO38141	(Pelot et al., 2017a)
IrCPS1v2	*n*-**L**-8(17)-ene	2°	Plant-c	*Isodon rubescens*	APJ36371	(Jin et al., 2017)
IrCPS2	*n*-**L**-8(17)-ene	2°	Plant-c	*Isodon rubescens*	APJ36372	(Jin et al., 2017)
NtLPS	*n*-**L**-8β-ol	2°	Plant-c	*Nicotiana tabacum*	G3CCC0	(Sallaud et al., 2012)
CcLPS	*n*-**L**-8β-ol	2°	Plant-c	*Cistus creticus*	ADJ93862	(Falara et al., 2010)
ApCPS1	*e&n*-**L**-8(17)-ene	2°	Plant-c	*Andrographis paniculata*	AXL95251	(Sun et al., 2019)
ArCPS1	*n*-**L**-8(17)-ene	2°	Plant-c	*Ajuga reptans*	AZB50377	(Johnson et al., 2019)
ArKPS	*ent*-**C**-4(18)-ene	2°	Plant-c	*Ajuga reptans*	AZB50378	(Johnson et al., 2019)
LlPPS	*syn*-**L**-9α-ol	2°	Plant-c	*Leonotis leonurus*	AZB50381	(Johnson et al., 2019)
MsCPS1	*n*-**L**-7-ene	2°	Plant-c	*Mesophaerum suaveolens*	AZB50380	(Johnson et al., 2019)
NrCPS1	*n*-**L**-8(17)-ene	2°	Plant-c	*Nepeta racemosa*	AZB50382	(Johnson et al., 2019)

OmCPS1	*n*-**L**-8(17)-ene	2°	Plant-c	*Origanum majorana*	AZB50383	(Johnson et al., 2019)
PcCPS1	*ent*-**L**-8-ene	2°	Plant-c	*Pogostemon cablin*	AZB50385	(Johnson et al., 2019)
SyCPS1	*n*-**L**-8(17)-ene	2°	Plant-c	*Salvia yangii*	AZB50384	(Johnson et al., 2019)
CaLPS	*ent*-**L**-8β-ol	2°	Plant-c	*Chiococca alba*	QIA61387	(Lau et al., 2020)
CamKPS	*ent*-**C**-3-ene	2°	Plant-c	*Callicarpa americana*	QMW69082	(Hamilton et al., 2020)
CamCPS6	*n*-**L**-8(17)-ene	2°	Plant-c	*Callicarpa americana*	QMW69083	(Hamilton et al., 2020)
CcCPS1	*n*-**L**-7-ene	2°	Plant-c	*Cistus creticus*	QZX44703	(Papanikolaou et al., 2024)
CcCPS2	*n*-**L**-7-ene	2°	Plant-c	*Cistus creticus*	QZX44704	(Papanikolaou et al., 2024)
IlCPS1	*n*-**L**-8(17)-ene	2°	Plant-c	*Isodon lophanthoides*	QXT24221	(Yang et al., 2021)
IlCPS2	*n*-**L**-7-ene	2°	Plant-c	*Isodon lophanthoides*	QXT24222	(Yang et al., 2021)
IlCPS3	*n*-**L**-8(17)-ene	2°	Plant-c	*Isodon lophanthoides*	QXT24223	(Yang et al., 2021)
CafCPS1	*ent*-**L**-8(17)-ene	2°	Plant-c	*Coffea arabica*	WMJ99068	(Ivamoto-Suzuki et al., 2023)
ZmCPS1	*ent*-**L**-8(17)-ene	GA	Plant-c	*Zea mays*	AAA73960	(Bensen et al., 1995)
OsCPS1	*ent*-**L**-8(17)-ene	GA	Plant-c	*Oryza sativa jap Nip*	NP_001403441	(Prisic et al., 2004)
TaCPS3	*ent*-**L**-8(17)-ene	GA	Plant-c	*Triticum aestivum*	BAH56560	(Toyomasu et al., 2009)
TaCPS4	*ent*-**L**-8(17)-ene	GA	Plant-c	*Triticum aestivum*	BAP01383	(Wu et al., 2012)
PvCPS14	*ent*-**L**-8(17)-ene	GA	Plant-c	*Panicum virgatum*	AXK78849	(Pelot et al., 2018)
SiTPS34	*ent*-**L**-8(17)-ene	GA	Plant-c	*Setaria italica*	QJA42363	(Karunanithi et al., 2020)
BdCPS	*ent*-**L**-8(17)-ene	GA/(LHT)	Plant-c	*Brachypodium distachyon*	BCK74095	(Shimada et al., 2023)
HvCPS1	*ent*-**L**-8(17)-ene	GA/(*R*)	Plant-c	*Hordeum vulgare*	AAT49065	(Wu et al., 2012)
ZmCPS2	*ent*-**L**-8(17)-ene	2°	Plant-c	*Zea mays*	AAT70083	(Harris et al., 2005)
ZmCPS3	*n*-**L**-8(17)-ene	2°	Plant-c	*Zea mays*	NP_001348098	(Murphy et al., 2018)
ZmCPS4	*n*-**L**-8-ene & β-ol	2°	Plant-c	*Zea mays*	XP_008680090	(Murphy et al., 2018)
OsCPS2	*ent*-**L**-8(17)-ene	2°	Plant-c	*Oryza sativa ind IR24*	AAT11021	(Prisic et al., 2004)
OsCPS4	*syn*-**L**-8(17)-ene	2°	Plant-c	*Oryza sativa ind IR24*	AAS98158	(Xu et al., 2004)
ObCPS2	*n*-**L**-8(17)-ene	2°	Plant-c	*Oryza brachyantha*	BAV31335	(Miyamoto et al., 2016)
HvCPS2	*n*-**L**-8(17)-ene	2°	Plant-c	*Hordeum vulgare*	BAJ95441	(Liu et al., 2021)
TaCPS1	*ent*-**L**-8(17)-ene	2°	Plant-c	*Triticum aestivum*	BAH56558	(Toyomasu et al., 2009)
TaCPS2	*n*-**L**-8(17)-ene	2°	Plant-c	*Triticum aestivum*	BAH56559	(Wu et al., 2012)
PvKPS1	*cis-syn*-**C**-3-ene	2°	Plant-c	*Panicum virgatum*	AXK78845	(Pelot et al., 2018)

PvCPS3	*n*-**L**-8-ene	2°	Plant-c	*Panicum virgatum*	AXK78846	(Pelot et al., 2018)
PvCPS8	*syn*-**L**-8(17)-ene	2°	Plant-c	*Panicum virgatum*	AXK78847	(Pelot et al., 2018)
PvCPS11	*ent*-**L**-8β-ol	2°	Plant-c	*Panicum virgatum*	AXK78848	(Pelot et al., 2018)
PvCPS15	*ent*-**L**-8(17)-ene	2°	Plant-c	*Panicum virgatum*	AXK78850	(Pelot et al., 2018)
SiTPS35	*ent*-**L**-8(17)-ene	2°	Plant-c	*Setaria italica*	QJA42364	(Karunanithi et al., 2020)
SiTPS6	*syn*-**L**-8(17)-ene	2°	Plant-c	*Setaria italica*	QJA42340	(Karunanithi et al., 2020)
SiTPS9	*n*-**L**-8(17)-ene	2°	Plant-c	*Setaria italica*	QJA42342	(Karunanithi et al., 2020)
BjCPS	*ent*-**L**-8(17)-ene	GA	Bact.	*Bradyrhizobium japonicum*	BAC47414	(Morrone et al., 2009)
MlCPS	*ent*-**L**-8(17)-ene	GA	Bact.	*Mesorhizobium loti*	WP_010913996	(Hershey et al., 2014)
SfCPS	*ent*-**L**-8(17)-ene	GA	Bact.	*Sinorhizobium fredii*	WP_010875301	(Hershey et al., 2014)
XoCPS	*ent*-**L**-8(17)-ene	GA	Bact.	*Xanthomonas oryzae*	WP_014501314	(Lu et al., 2015)
XtCPS	*ent*-**L**-8(17)-ene	GA	Bact.	*Xanthomonas translucens*	CCP42266	(Nagel and Peters, 2017)
PmCPS	*ent*-**L**-8(17)-ene	GA	Bact.	*Paraburkholderia mimosarum*	WP_208461029	(Nagel et al., 2018)
AaCPS	*ent*-**L**-8(17)-ene	GA	Bact.	*Acidovorax avenae*	WP_208934609	(Ogonkov et al., 2023)
ReCPS	*ent*- **L**-8β-ol + CPP	GA/(PSV)	Bact.	*Rhizobium etli*	WP_011053441	(Hershey et al., 2014)
KgTPS	*syn*-**C**-3-ene	2°	Bact.	*Kitasatospora griseola*	BAB39206	(Dairi et al., 2001)
SsCPS	*e*/*n*-**L**-8(17)-ene	2°	Bact.	*Streptomyces* sp. KO-3988	BAD86797	(Kawasaki et al., 2004)
MtHPS	*n*-**H**-5-ene	2°	Bact.	*Mycobacterium tuberculosis*	WP_003417905	(Nakano et al., 2005)
SpCPS	*ent*-**L**-8(17)-ene	2°	Bact.	*Streptomyces platensis*	ACO31276	(Smanski et al., 2011)
SaCPS	*n*-**L**-8(17)-ene	2°	Bact.	*Salinispora arenicola*	WP_018908571	(Xu et al., 2014)
SgCPS	*e*/*n*-**L**-8(17)-ene	2°	Bact.	*Streptomyces griseus*	AHK61132	(Xie et al., 2014)
ScCPS	*e*/*n*-**L**-8(17)-ene	2°	Bact.	*Streptomyces cyslabdanicus*	BAR97451	(Ikeda et al., 2016)
SanCPS	*e*/*n*-**L**-8(17)-ene	2°	Bact.	*Streptomyces anulatus*	BAR97461	(Ikeda et al., 2016)
SclCPS	*e*/*n*-**L**-8(17)-ene	2°	Bact.	*Streptomyces clavuligerus*	WP_003963126	(Yamada et al., 2016)
HaKPS	*n*-**C**-3-ene	2°	Bact.	*Herpetosiphon aurantiacus*	A9AWD5	(Nakano et al., 2015)
SkCPS	*e*/*n*-**L**-8(17)-ene	2°	Bact.	*Streptomyces* sp. K155	WP_086020186	(Centeno-Leija et al., 2019)
KsTPS	*syn*-**C**-3-ene	2°	Bact.	*Kitasatospora* sp. CB02891	PJN29585	(Stowell et al., 2022)
StCPS	*syn*-**L**-8(17)-ene	2°	Bact.	*Scytonema tolypothrichoides*	KAB8331911	(Yu et al., 2025)

CsCPSKS	*ent*-**L**-8(17)-ene	2°	Bact.	*Candidatus sericytochromatia*	MBC7542232	(Chen et al., 2024)
CjCPSPS	*n*-**L**-8(17)-ene	2°	Bact.	*Chitinophaga japonensis*	WP_145718914	(Chen et al., 2024)
SsCPSAS	*syn*-**L**-8(17)-ene	2°	Bact.	*Streptomyces* sp. GS7	WP_159504149	(Chen et al., 2024)
GfCPSKS	*ent*-**L**-8(17)-ene	GA	Fungi	*Gibberella fujikuroi*	CAA75244	(Tudzynski et al., 1998)
PsCPSKS	*ent*-**L**-8(17)-ene	GA	Fungi	*Phaeosphaeria* sp. L487	BAA22426	(Kawaide et al., 1997)
SmCPSKS	*ent*-**L**-8(17)-ene	GA	Fungi	*Sphaceloma manihoticola*	CAP07655	(Bomke et al., 2008)
FpCPSKS	*ent*-**L**-8(17)-ene	GA	Fungi	*Fusarium proliferatum*	CAP74389	(Tsavkelova et al., 2008)
PbCPSAS	*syn*-**2**-8(17)-ene	2°	Fungi	*Phoma betae*	BAB62102	(Oikawa et al., 2001)
PaCPSPS	*n*-**L**-8(17)-ene	2°	Fungi	*Phomopsis amygdali*	BAG30961	(Toyomasu et al., 2008)
PaCPS	*n*-**L**-8(17)-ene	2°	Fungi	*Phomopsis amygdali*	BAG30962	(Toyomasu et al., 2008)
AfCPSPS	*n*-**L**-8(17)-ene	2°	Fungi	*Aspergillus fumigatus*	XP_753151	(Xu et al., 2017)
AoCPSPS	*n*-**L**-8(17)-ene	2°	Fungi	*Aspergillus oryzae*	XP_001820661	(Xu et al., 2017)
AnCPSPS	*n*-**L**-8(17)-ene	2°	Fungi	*Aspergillus niger*	XP_001398730	(Xu et al., 2017)
NfCPSPS	*n*-**L**-8(17)-ene	2°	Fungi	*Neosartorya fischeri*	XP_001264196	(Xu et al., 2017)
CpPS	MPP	2°	Fungi	*Clitopilus passeckerianus*	AVA16672	(Xu et al., 2018)
RpPS	MPP	2°	Fungi	*Rhodocybe pseudopiperita*	A0A6S6QR11	(Yamane et al., 2017)
MoDTPS1	*ent*-**L**-8β-ol	2°	Fungi	*Magnaporthe oryzae*	XP_003708691	(Shahi et al., 2022)
MoDTPS2	*n*-**L**-8(17)-ene	2°	Fungi	*Magnaporthe oryzae*	XP_003719120	(Shahi et al., 2022)

[1]Plant DTCs are further sub-divided by terpene synthase sub-family (Chen et al., 2011).

[2]GA/(indicates variation defined here as leaving role in GA biosynthesis uncertain, either substitution for regulatory *H* (e.g., *R*) or in catalytic base dyad motifs – highlighted residue).

Figure S2: Effect of all described mutants on product outcome.

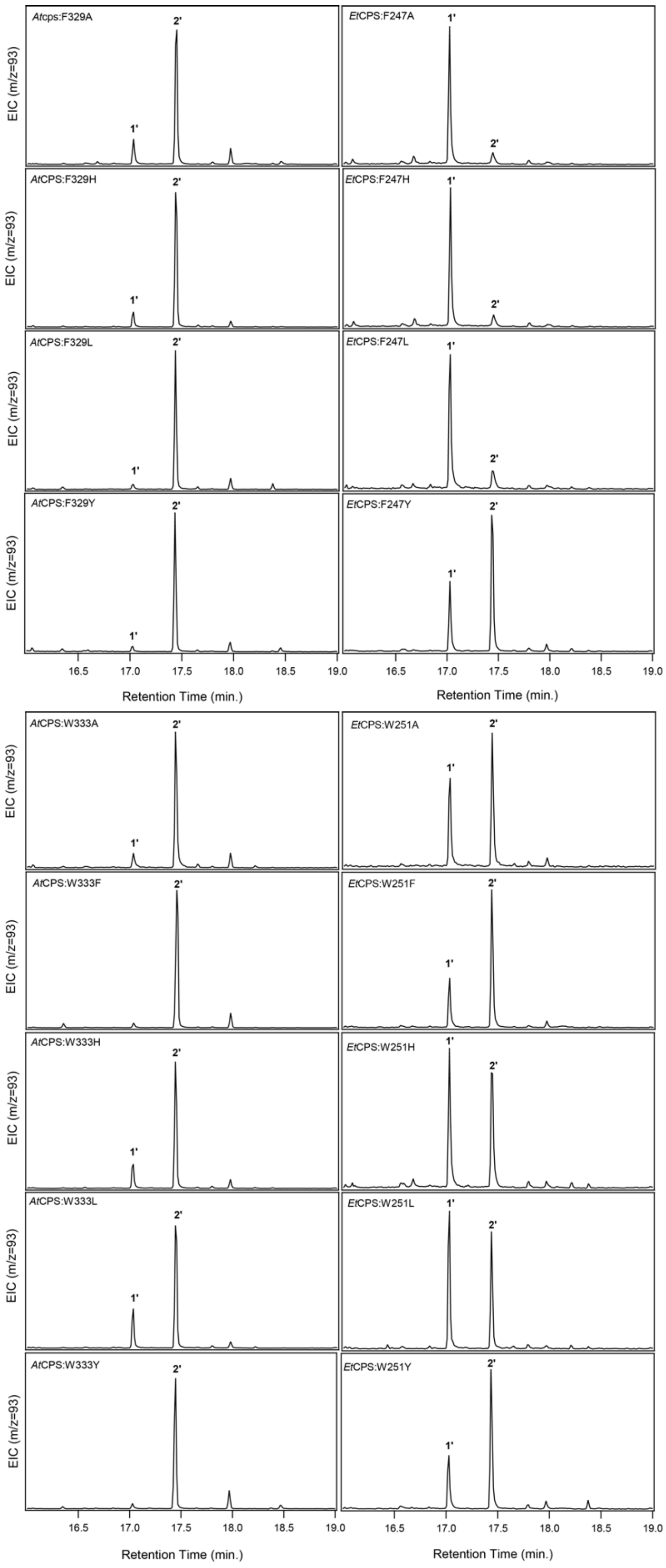

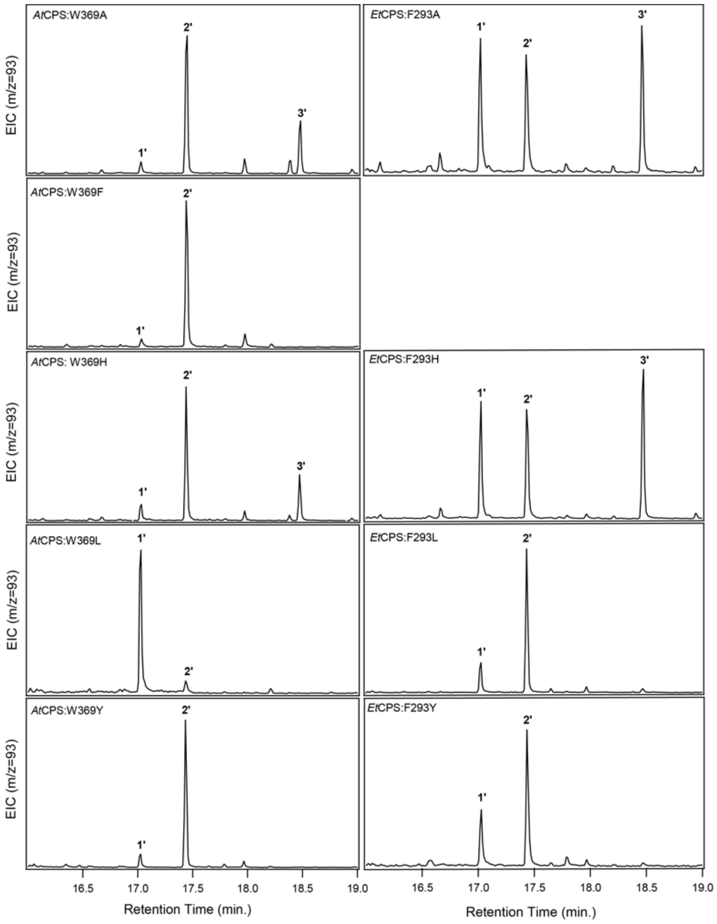
AtCPS:W369A
EtCPS:F293A
AtCPS:W369F
AtCPS: W369H
EtCPS:F293H
AtCPS:W369L
EtCPS:F293L
AtCPS:W369Y
EtCPS:F293Y
EIC (m/z=93)
1'
2'
3'
16.5
17.0
17.5
18.0
18.5
19.0
Retention Time (min.)

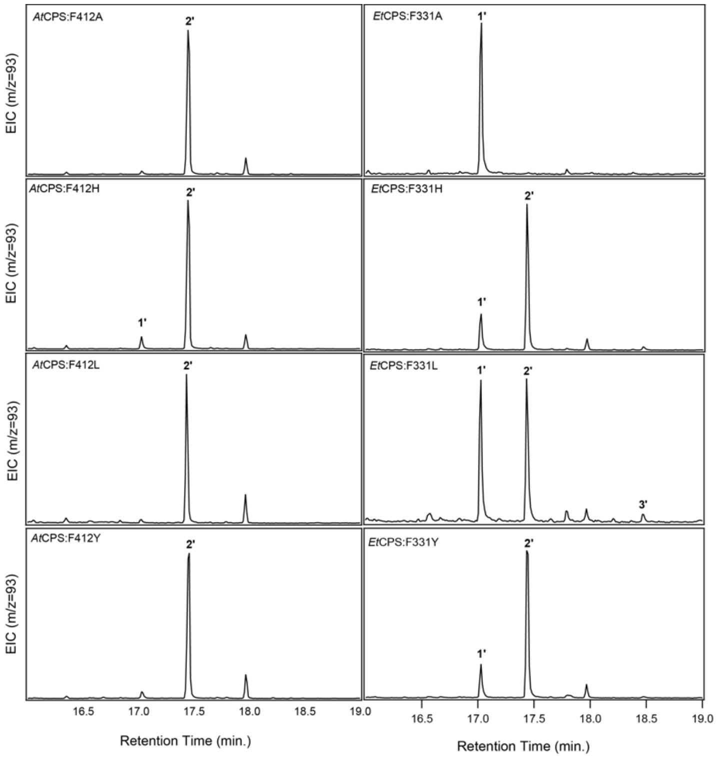
AtCPS:F412A
EtCPS:F331A
AtCPS:F412H
EtCPS:F331H
AtCPS:F412L
EtCPS:F331L
AtCPS:F412Y
EtCPS:F331Y
EIC (m/z=93)
1'
2'
3'
16.5
17.0
17.5
18.0
18.5
19.0
Retention Time (min.)

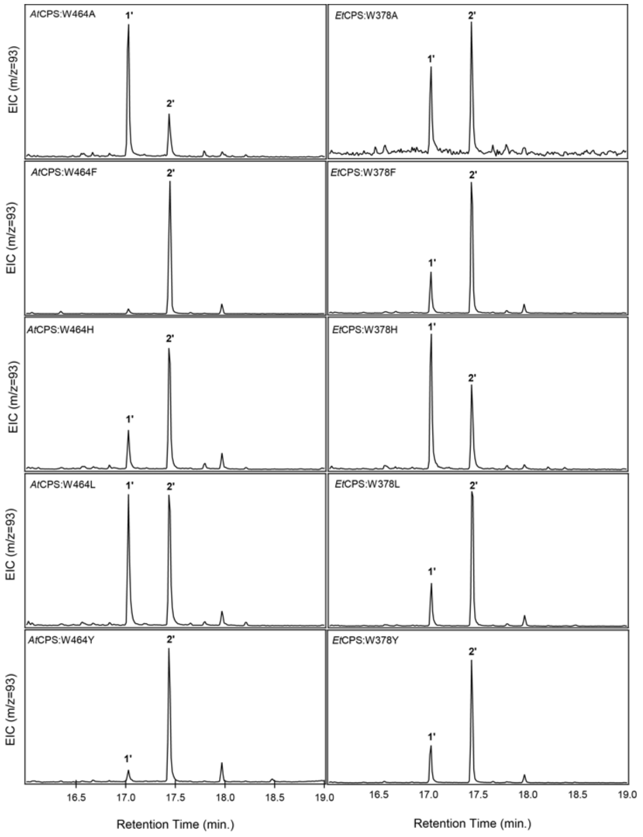
AtCPS:W464A
AtCPS:W464F
AtCPS:W464H
AtCPS:W464L
AtCPS:W464Y
EtCPS:W378A
EtCPS:W378F
EtCPS:W378H
EtCPS:W378L
EtCPS:W378Y
1'
2'
EIC (m/z=93)
16.5
17.0
17.5
18.0
18.5
19.0
Retention Time (min.)

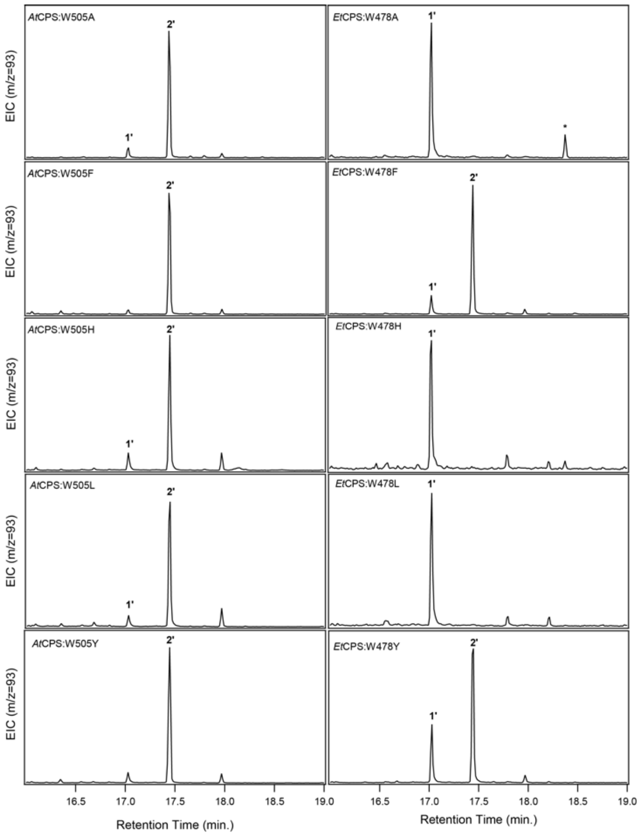
AtCPS:W505A
AtCPS:W505F
AtCPS:W505H
AtCPS:W505L
AtCPS:W505Y
EtCPS:W478A
EtCPS:W478F
EtCPS:W478H
EtCPS:W478L
EtCPS:W478Y
1'
2'
*
EIC (m/z=93)
16.5
17.0
17.5
18.0
18.5
19.0
Retention Time (min.)

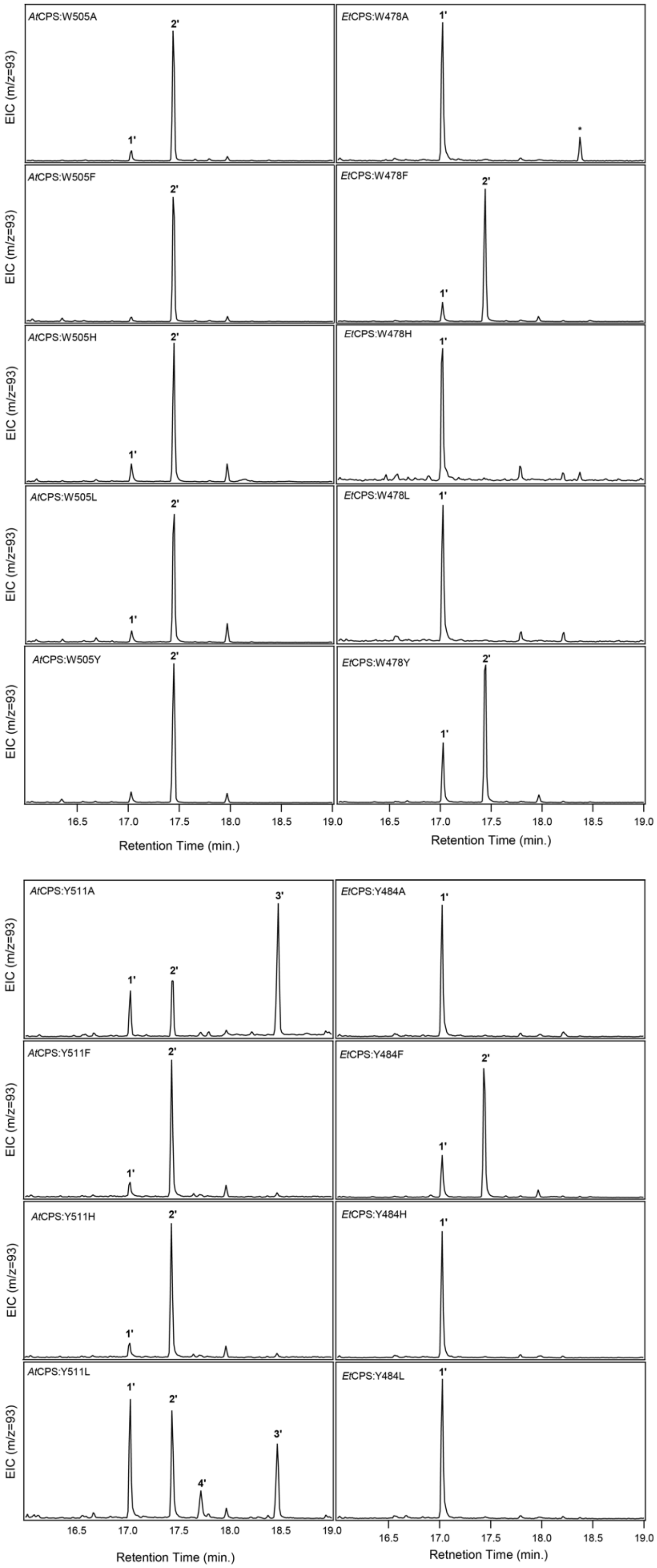
AtCPS:W505A
EtCPS:W478A
AtCPS:W505F
EtCPS:W478F
AtCPS:W505H
EtCPS:W478H
AtCPS:W505L
EtCPS:W478L
AtCPS:W505Y
EtCPS:W478Y
AtCPS:Y511A
EtCPS:Y484A
AtCPS:Y511F
EtCPS:Y484F
AtCPS:Y511H
EtCPS:Y484H
AtCPS:Y511L
EtCPS:Y484L
EIC (m/z=93)
Retention Time (min.)
Retnetion Time (min.)
1'
2'
3'
4'
*

References

Ait-Ali, T., Swain, S. M., Reid, J. B., Sun, T., Kamiya, Y., 1997. The LS locus of pea encodes the gibberellin biosynthesis enzyme *ent*-kaurene synthase A. Plant J 11, 443-454. https://doi.org/10.1046/j.1365-313x.1997.11030443.x.

Andersen-Ranberg, J., Kongstad, K. T., Nielsen, M. T., Jensen, N. B., Pateraki, I., Bach, S. S., Hamberger, B., Zerbe, P., Staerk, D., Bohlmann, J., Moller, B. L., Hamberger, B., 2016. Expanding the Landscape of Diterpene Structural Diversity through Stereochemically Controlled Combinatorial Biosynthesis. Angew Chem Int Ed Engl 55, 2142-2146. https://doi.org/10.1002/anie.201510650.

Bensen, R. J., Johal, G. S., Crane, V. C., Tossberg, J. T., Schnable, P. S., Meeley, R. B., Briggs, S. P., 1995. Cloning and characterization of the maize An1 gene. Plant Cell 7, 75-84. https://doi.org/10.1105/tpc.7.1.75.

Bomke, C., Rojas, M. C., Gong, F., Hedden, P., Tudzynski, B., 2008. Isolation and characterization of the gibberellin biosynthetic gene cluster in Sphaceloma manihoticola. Appl. Environ. Microbiol. 74, 5325-5339. https://doi.org/10.1128/AEM.00694-08.

Bozic, D., Papaefthimiou, D., Bruckner, K., de Vos, R. C., Tsoleridis, C. A., Katsarou, D., Papanikolaou, A., Pateraki, I., Chatzopoulou, F. M., Dimitriadou, E., Kostas, S., Manzano, D., Scheler, U., Ferrer, A., Tissier, A., Makris, A. M., Kampranis, S. C., Kanellis, A. K., 2015. Towards Elucidating Carnosic Acid Biosynthesis in Lamiaceae: Functional Characterization of the Three First Steps of the Pathway in Salvia fruticosa and Rosmarinus officinalis. PLoS One 10, e0124106. https://doi.org/10.1371/journal.pone.0124106.

Caniard, A., Zerbe, P., Legrand, S., Cohade, A., Valot, N., Magnard, J. L., Bohlmann, J., Legendre, L., 2012. Discovery and functional characterization of two diterpene synthases for sclareol biosynthesis in *Salvia sclarea* (L.) and their relevance for perfume manufacture. BMC Plant Biol. 12, 119. https://doi.org/10.1186/1471-2229-12-119.

Centeno-Leija, S., Tapia-Cabrera, S., Guzman-Trampe, S., Esquivel, B., Esturau-Escofet, N., Tierrafria, V. H., Rodriguez-Sanoja, R., Zarate-Romero, A., Stojanoff, V., Rudino-Pinera, E., Sanchez, S., Serrano-Posada, H., 2019. The structure of (E)-biformene synthase provides insights into the biosynthesis of bacterial bicyclic labdane-related diterpenoids. J Struct Biol 207, 29-39. https://doi.org/10.1016/j.jsb.2019.04.010.

Chen, F., Tholl, D., Bohlmann, J., Pichersky, E., 2011. The family of terpene synthases in plants: a mid-size family of genes for specialized metabolism that is highly diversified throughout the kingdom. Plant J 66, 212-229. https://doi.org/10.1111/j.1365-313X.2011.04520.x.

Chen, X., Berim, A., Dayan, F. E., Gang, D. R., 2017. A (-)-kolavenyl diphosphate synthase catalyzes the first step of salvinorin A biosynthesis in Salvia divinorum. J Exp Bot 68, 1109-1122. https://doi.org/10.1093/jxb/erw493.

Chen, X., Xu, M., Han, J., Schmidt-Dannert, M., Peters, R. J., Chen, F., 2024. Discovery of bifunctional diterpene cyclases/synthases in bacteria supports a bacterial origin for the plant terpene synthase gene family. Hortic Res 11, uhae221. https://doi.org/10.1093/hr/uhae221.
Cui, G., Duan, L., Jin, B., Qian, J., Xue, Z., Shen, G., Snyder, J. H., Song, J., Chen, S., Huang, L., Peters, R. J., Qi, X., 2015. Functional Divergence of Diterpene Syntheses in the Medicinal Plant Salvia miltiorrhiza. Plant Physiol 169, 1607-1618. https://doi.org/10.1104/pp.15.00695.
Dairi, T., Hamano, Y., Kuzuyama, T., Itoh, N., Furihata, K., Seto, H., 2001. Eubacterial diterpene cyclase genes essential for production of the isoprenoid antibiotic terpentecin. J Bacteriol 183, 6085-6094. https://doi.org/10.1128/JB.183.20.6085-6094.2001.
Falara, V., Pichersky, E., Kanellis, A. K., 2010. A copal-8-ol diphosphate synthase from the angiosperm *Cistus creticus* subsp. *creticus* is a putative key enzyme for the formation of pharmacologically active, oxygen-containing labdane-type diterpenes. Plant Physiol 154, 301-310. https://doi.org/10.1104/pp.110.159566.
Gao, W., Hillwig, M. L., Huang, L., Cui, G., Wang, X., Kong, J., Yang, B., Peters, R. J., 2009. A functional genomics approach to tanshinone biosynthesis provides stereochemical insights. Org Lett 11, 5170-5173. https://doi.org/10.1021/ol902051v.
Hall, D. E., Zerbe, P., Jancsik, S., Quesada, A. L., Dullat, H., Madilao, L. L., Yuen, M., Bohlmann, J., 2013. Evolution of conifer diterpene synthases: diterpene resin acid biosynthesis in lodgepole pine and jack pine involves monofunctional and bifunctional diterpene synthases. Plant Physiol 161, 600-616. https://doi.org/10.1104/pp.112.208546.
Hamilton, J. P., Godden, G. T., Lanier, E., Bhat, W. W., Kinser, T. J., Vaillancourt, B., Wang, H., Wood, J. C., Jiang, J., Soltis, P. S., Soltis, D. E., Hamberger, B., Buell, C. R., 2020. Generation of a chromosome-scale genome assembly of the insect-repellent terpenoid-producing Lamiaceae species, Callicarpa americana. Gigascience 9 https://doi.org/10.1093/gigascience/giaa093.
Hansen, N. L., Heskes, A. M., Hamberger, B., Olsen, C. E., Hallstrom, B. M., Andersen-Ranberg, J., Hamberger, B., 2017. The terpene synthase gene family in Tripterygium wilfordii harbors a labdane-type diterpene synthase among the monoterpene synthase TPS-b subfamily. Plant J 89, 429-441. https://doi.org/10.1111/tpj.13410.
Harris, L. J., Saparno, A., Johnston, A., Prisic, S., Xu, M., Allard, S., Kathiresan, A., Ouellet, T., Peters, R. J., 2005. The maize An2 gene is induced by Fusarium attack and encodes an ent-copalyl diphosphate synthase. Plant Mol Biol 59, 881-894. https://doi.org/10.1007/s11103-005-1674-8.
Hayashi, K., Kawaide, H., Notomi, M., Sakigi, Y., Matsuo, A., Nozaki, H., 2006. Identification and functional analysis of bifunctional ent-kaurene synthase from the moss *Physcomitrella patens*. FEBS Lett 580, 6175-6181. https://doi.org/10.1016/j.febslet.2006.10.018.
Hershey, D. M., Lu, X., Zi, J., Peters, R. J., 2014. Functional conservation of the capacity for *ent*-kaurene biosynthesis and an associated operon in certain rhizobia. J Bacteriol 196, 100-106. https://doi.org/10.1128/JB.01031-13.

Heskes, A. M., Sundram, T. C. M., Boughton, B. A., Jensen, N. B., Hansen, N. L., Crocoll, C., Cozzi, F., Rasmussen, S., Hamberger, B., Hamberger, B., Staerk, D., Moller, B. L., Pateraki, I., 2018. Biosynthesis of bioactive diterpenoids in the medicinal plant Vitex agnus-castus. Plant J 93, 943-958. https://doi.org/10.1111/tpj.13822.
Ikeda, H., Shin-Ya, K., Nagamitsu, T., Tomoda, H., 2016. Biosynthesis of mercapturic acid derivative of the labdane-type diterpene, cyslabdan that potentiates imipenem activity against methicillin-resistant Staphylococcus aureus: cyslabdan is generated by mycothiol-mediated xenobiotic detoxification. J Ind Microbiol Biotechnol 43, 325-342. https://doi.org/10.1007/s10295-015-1694-6.
Inabuy, F., Fischedick, J. T., Lange, I., Hartmann, M., Srividya, N., Parrish, A. N., Xu, M., Peters, R. J., Lange, B. M., 2017. Biosynthesis of Diterpenoids in Tripterygium Adventitious Root Cultures. Plant Physiol 175, 92-103. https://doi.org/10.1104/pp.17.00659.
Irmisch, S., Muller, A. T., Schmidt, L., Gunther, J., Gershenzon, J., Kollner, T. G., 2015. One amino acid makes the difference: the formation of ent-kaurene and 16alpha-hydroxy-ent-kaurane by diterpene synthases in poplar. BMC Plant Biol 15, 262. https://doi.org/10.1186/s12870-015-0647-6.
Ivamoto-Suzuki, S. T., Celedon, J. M., Yuen, M. M. S., Kitzberger, C. S. G., Silva Domingues, D., Bohlmann, J., Protasio Pereira, L. F., 2023. Functional Characterization of ent-Copalyl Diphosphate Synthase and Kaurene Synthase Genes from Coffea arabica L. J Agric Food Chem 71, 15863-15873. https://doi.org/10.1021/acs.jafc.2c09087.
Jia, Q., Brown, R., Kollner, T. G., Fu, J., Chen, X., Wong, G. K., Gershenzon, J., Peters, R. J., Chen, F., 2022. Origin and Early Evolution of the Plant Terpene Synthase Family. Proc Natl Acad Sci U S A 119, e2100361119. https://doi.org/10.1073/pnas.2100361119.
Jin, B., Cui, G., Guo, J., Tang, J., Duan, L., Lin, H., Shen, Y., Chen, T., Zhang, H., Huang, L., 2017. Functional Diversification of Kaurene Synthase-Like Genes in Isodon rubescens. Plant Physiol 174, 943-955. https://doi.org/10.1104/pp.17.00202.
Johnson, S. R., Bhat, W. W., Bibik, J., Turmo, A., Hamberger, B., Evolutionary Mint Genomics, C., Hamberger, B., 2019. A database-driven approach identifies additional diterpene synthase activities in the mint family (Lamiaceae). J Biol Chem 294, 1349-1362. https://doi.org/10.1074/jbc.RA118.006025.
Karunanithi, P. S., Berrios, D. I., Wang, S., Davis, J., Shen, T., Fiehn, O., Maloof, J. N., Zerbe, P., 2020. The foxtail millet (Setaria italica) terpene synthase gene family. Plant J 103, 781-800. https://doi.org/10.1111/tpj.14771.
Kawaide, H., Hayashi, K., Kawanabe, R., Sakigi, Y., Matsuo, A., Natsume, M., Nozaki, H., 2011. Identification of the single amino acid involved in quenching the *ent*-kauranyl cation by a water molecule in *ent*-kaurene synthase of *Physcomitrella patens*. FEBS J. 278, 123-133. https://doi.org/10.1111/j.1742-4658.2010.07938.x.

Kawaide, H., Imai, R., Sassa, T., Kamiya, Y., 1997. *Ent*-kaurene synthase from the fungus *Phaeosphaeria* sp. L487. cDNA isolation, characterization, and bacterial expression of a bifunctional diterpene cyclase in fungal gibberellin biosynthesis. J Biol Chem 272, 21706-21712. https://doi.org/10.1074/jbc.272.35.21706.

Kawasaki, T., Kuzuyama, T., Kuwamori, Y., Matsuura, N., Itoh, N., Furihata, K., Seto, H., Dairi, T., 2004. Presence of copalyl diphosphate synthase gene in an actinomycete possessing the mevalonate pathway. The Journal of antibiotics 57, 739-747. https://doi.org/10.7164/antibiotics.57.739.

Keeling, C. I., Dullat, H. K., Yuen, M., Ralph, S. G., Jancsik, S., Bohlmann, J., 2010. Identification and functional characterization of monofunctional *ent*-copalyl diphosphate and *ent*-kaurene synthases in white spruce reveal different patterns for diterpene synthase evolution for primary and secondary metabolism in gymnosperms. Plant Physiol 152, 1197-1208. https://doi.org/10.1104/pp.109.151456.

Keeling, C. I., Weisshaar, S., Ralph, S. G., Jancsik, S., Hamberger, B., Dullat, H. K., Bohlmann, J., 2011. Transcriptome mining, functional characterization, and phylogeny of a large terpene synthase gene family in spruce (*Picea* spp.). BMC Plant Biol 11, 43. https://doi.org/10.1186/1471-2229-11-43.

Kim, M. J., Jin, J., Zheng, J., Wong, L., Chua, N. H., Jang, I. C., 2015. Comparative Transcriptomics Unravel Biochemical Specialization of Leaf Tissues of Stevia for Diterpenoid Production. Plant Physiol 169, 2462-2480. https://doi.org/10.1104/pp.15.01353.

Kumar, S., Kempinski, C., Zhuang, X., Norris, A., Mafu, S., Zi, J., Bell, S. A., Nybo, S. E., Kinison, S. E., Jiang, Z., Goklany, S., Linscott, K. B., Chen, X., Jia, Q., Brown, S. D., Bowman, J. L., Babbitt, P. C., Peters, R. J., Chen, F., Chappell, J., 2016. Molecular Diversity of Terpene Synthases in the Liverwort Marchantia polymorpha. Plant Cell 28, 2632-2650. https://doi.org/10.1105/tpc.16.00062.

Lau, K. H., Bhat, W. W., Hamilton, J. P., Wood, J. C., Vaillancourt, B., Wiegert-Rininger, K., Newton, L., Hamberger, B., Holmes, D., Hamberger, B., Buell, C. R., 2020. Genome assembly of Chiococca alba uncovers key enzymes involved in the biosynthesis of unusual terpenoids. DNA Res 27 https://doi.org/10.1093/dnares/dsaa013.

Li, G., Kollner, T. G., Yin, Y., Jiang, Y., Chen, H., Xu, Y., Gershenzon, J., Pichersky, E., Chen, F., 2012a. Nonseed plant *Selaginella moellendorffi* has both seed plant and microbial types of terpene synthases. Proc Natl Acad Sci U S A 109, 14711-14715. https://doi.org/10.1073/pnas.1204300109.

Li, J. L., Chen, Q. Q., Jin, Q. P., Gao, J., Zhao, P. J., Lu, S., Zeng, Y., 2012b. IeCPS2 is potentially involved in the biosynthesis of pharmacologically active Isodon diterpenoids rather than gibberellin. Phytochemistry 76, 32-39. https://doi.org/10.1016/j.phytochem.2011.12.021.

Liu, Y., Balcke, G. U., Porzel, A., Mahdi, L., Scherr-Henning, A., Bathe, U., Zuccaro, A., Tissier, A., 2021. A barley gene cluster for the biosynthesis of diterpenoid phytoalexins. bioRxiv https://doi.org/https://doi.org/10.1101/2021.05.21.445084.
Lu, X., Hershey, D. M., Wang, L., Bogdanove, A. J., Peters, R. J., 2015. An ent-kaurene-derived diterpenoid virulence factor from Xanthomonas oryzae pv. oryzicola. New Phytol 206, 295-302. https://doi.org/10.1111/nph.13187.
Ma, L. T., Lee, Y. R., Tsao, N. W., Wang, S. Y., Zerbe, P., Chu, F. H., 2019. Biochemical characterization of diterpene synthases of Taiwania cryptomerioides expands the known functional space of specialized diterpene metabolism in gymnosperms. Plant J 100, 1254-1272. https://doi.org/10.1111/tpj.14513.
Ma, L. T., Wang, C. H., Hon, C. Y., Lee, Y. R., Chu, F. H., 2021. Discovery and characterization of diterpene synthases in Chamaecyparis formosensis Matsum. which participated in an unprecedented diterpenoid biosynthesis route in conifer. Plant Sci 304, 110790. https://doi.org/10.1016/j.plantsci.2020.110790.
Mafu, S., Hillwig, M. L., Peters, R. J., 2011. A novel labda-7,13e-dien-15-ol-producing bifunctional diterpene synthase from *Selaginella moellendorffii*. ChemBioChem 12, 1984-1987. https://doi.org/10.1002/cbic.201100336.
Martin, D. M., Faldt, J., Bohlmann, J., 2004. Functional characterization of nine Norway Spruce TPS genes and evolution of gymnosperm terpene synthases of the TPS-d subfamily. Plant Physiol 135, 1908-1927. https://doi.org/10.1104/pp.104.042028.
Misra, R. C., Garg, A., Roy, S., Chanotiya, C. S., Vasudev, P. G., Ghosh, S., 2015. Involvement of an *ent*-copalyl diphosphate synthase in tissue-specific accumulation of specialized diterpenes in *Andrographis paniculata*. Plant Sci 240, 50-64. https://doi.org/10.1016/j.plantsci.2015.08.016.
Miyamoto, K., Fujita, M., Shenton, M. R., Akashi, S., Sugawara, C., Sakai, A., Horie, K., Hasegawa, M., Kawaide, H., Mitsuhashi, W., Nojiri, H., Yamane, H., Kurata, N., Okada, K., Toyomasu, T., 2016. Evolutionary trajectory of phytoalexin biosynthetic gene clusters in rice. Plant J 87, 293-304. https://doi.org/10.1111/tpj.13200.
Morrone, D., Chambers, J., Lowry, L., Kim, G., Anterola, A., Bender, K., Peters, R. J., 2009. Gibberellin biosynthesis in bacteria: Separate *ent*-copalyl diphosphate and *ent*-kaurene synthases in *Bradyrhizobium japonicum*. FEBS Lett. 583, 475-480. https://doi.org/10.1016/j.febslet.2008.12.052.
Murphy, K. M., Ma, L. T., Ding, Y., Schmelz, E. A., Zerbe, P., 2018. Functional Characterization of Two Class II Diterpene Synthases Indicates Additional Specialized Diterpenoid Pathways in Maize (Zea mays). Front Plant Sci 9, 1542. https://doi.org/10.3389/fpls.2018.01542.
Nagel, R., Bieber, J., Schmidt-Dannert, M. G., Nett, R. S., Peters, R. J., 2018. A third class: Functional gibberellin biosynthetic operon in beta-proteobacteria. Front Microbiol 9, 2916. https://doi.org/10.3389/fmicb.2018.02916.

Nagel, R., Peters, R. J., 2017. Investigating the Phylogenetic Range of Gibberellin Biosynthesis in Bacteria. Mol Plant Microbe Interact 30, 343-349. https://doi.org/10.1094/MPMI-01-17-0001-R.

Nakagiri, T., Lee, J.-B., Hayashi, T., 2005. cDNA cloning, functional expression and characterization of *ent*-copalyl diphosphate synthase from *Scoparia dulcis* L. Plant Sci 169, 760-767. https://doi.org/10.1016/j.plantsci.2005.05.028.

Nakano, C., Okamura, T., Sato, T., Dairi, T., Hoshino, T., 2005. Mycobacterium tuberculosis H37Rv3377c encodes the diterpene cyclase for producing the halimane skeleton. Chem Commun (Camb) 2005, 1016-1018. https://doi.org/10.1039/b415346d.

Nakano, C., Oshima, M., Kurashima, N., Hoshino, T., 2015. Identification of a new diterpene biosynthetic gene cluster that produces O-methylkolavelool in Herpetosiphon aurantiacus. Chembiochem 16, 772-781. https://doi.org/10.1002/cbic.201402652.

Ogonkov, A., Brosius, P. E., Zeng, Q., Sasso, S., Nagel, R., 2023. Not All Acidovorax Are Created Equal: Gibberellin Biosynthesis in the Turfgrass Pathogen Acidovorax avenae subsp. avenae. Mol Plant Microbe Interact 36, 647-655. https://doi.org/10.1094/MPMI-02-23-0017-R.

Oikawa, H., Toyomasu, T., Toshima, H., Ohashi, S., Kawaide, H., Kamiya, Y., Ohtsuka, M., Shinoda, S., Mitsuhashi, W., Sassa, T., 2001. Cloning and functional expression of cDNA encoding aphidicolan-16 beta-ol synthase: a key enzyme responsible for formation of an unusual diterpene skeleton in biosynthesis of aphidicolin. J Am Chem Soc 123, 5154-5155. https://doi.org/10.1021/ja015747j.

Okada, K., Kawaide, H., Miyamoto, K., Miyazaki, S., Kainuma, R., Kimura, H., Fujiwara, K., Natsume, M., Nojiri, H., Nakajima, M., Yamane, H., Hatano, Y., Nozaki, H., Hayashi, K., 2016. HpDTC1, a Stress-Inducible Bifunctional Diterpene Cyclase Involved in Momilactone Biosynthesis, Functions in Chemical Defence in the Moss Hypnum plumaeforme. Sci Rep 6, 25316. https://doi.org/10.1038/srep25316.

Papanikolaou, A. S., Papaefthimiou, D., Matekalo, D., Karakousi, C. V., Makris, A. M., Kanellis, A. K., 2024. Chemical and transcriptomic analyses of leaf trichomes from Cistus creticus subsp. creticus reveal the biosynthetic pathways of certain labdane-type diterpenoids and their acetylated forms. J Exp Bot 75, 3431-3451. https://doi.org/10.1093/jxb/erae098.

Pateraki, I., Andersen-Ranberg, J., Hamberger, B., Heskes, A. M., Martens, H. J., Zerbe, P., Bach, S. S., Moller, B. L., Bohlmann, J., Hamberger, B., 2014. Manoyl Oxide (13R), the Biosynthetic Precursor of Forskolin, Is Synthesized in Specialized Root Cork Cells in Coleus forskohlii. Plant Physiol 164, 1222-1236. https://doi.org/10.1104/pp.113.228429.

Pelot, K. A., Chen, R., Hagelthorn, D. M., Young, C. A., Addison, J. B., Muchlinski, A., Tholl, D., Zerbe, P., 2018. Functional Diversity of Diterpene Synthases in the Biofuel Crop Switchgrass. Plant Physiol 178, 54-71. https://doi.org/10.1104/pp.18.00590.

Pelot, K. A., Hagelthorn, D. M., Addison, J. B., Zerbe, P., 2017a. Biosynthesis of the oxygenated diterpene nezukol in the medicinal plant Isodon rubescens is catalyzed by a pair of diterpene synthases. PLoS One 12, e0176507. https://doi.org/10.1371/journal.pone.0176507.

Pelot, K. A., Mitchell, R., Kwon, M., Hagelthorn, D. M., Wardman, J. F., Chiang, A., Bohlmann, J., Ro, D. K., Zerbe, P., 2017b. Biosynthesis of the psychotropic plant diterpene salvinorin A: Discovery and characterization of the Salvia divinorum clerodienyl diphosphate synthase. Plant J 89, 885-897. https://doi.org/10.1111/tpj.13427.
Prisic, S., Xu, M., Wilderman, P. R., Peters, R. J., 2004. Rice contains two disparate *ent*-copalyl diphosphate synthases with distinct metabolic functions. Plant Physiol. 136, 4228-4236. https://doi.org/10.1104/pp.104.050567.
Rebers, M., Kaneta, T., Kawaide, H., Yamaguchi, S., Yang, Y. Y., Imai, R., Sekimoto, H., Kamiya, Y., 1999. Regulation of gibberellin biosynthesis genes during flower and early fruit development of tomato. Plant J 17, 241-250. https://doi.org/10.1046/j.1365-313x.1999.00366.x.
Richman, A. S., Gijzen, M., Starratt, A. N., Yang, Z., Brandle, J. E., 1999. Diterpene synthesis in *Stevia rebaudiana*: recruitment and up-regulation of key enzymes from the gibberellin biosynthetic pathway. Plant J 19, 411-421. https://doi.org/10.1046/j.1365-313x.1999.00531.x.
Ro, D. K., Bohlmann, J., 2006. Diterpene resin acid biosynthesis in loblolly pine (*Pinus taeda*): functional characterization of abietadiene/levopimaradiene synthase (PtTPS-LAS) cDNA and subcellular targeting of PtTPS-LAS and abietadienol/abietadienal oxidase (PtAO, CYP720B1). Phytochemistry 67, 1572-1578. https://doi.org/10.1016/j.phytochem.2006.01.011.
Sallaud, C., Giacalone, C., Topfer, R., Goepfert, S., Bakaher, N., Rosti, S., Tissier, A., 2012. Characterization of two genes for the biosynthesis of the labdane diterpene Z-abienol in tobacco (Nicotiana tabacum) glandular trichomes. Plant J. 72, 1-17. https://doi.org/10.1111/j.1365-313X.2012.05068.x.
Sawada, Y., Katsumata, T., Kitamura, J., Kawaide, H., Nakajima, M., Asami, T., Nakaminami, K., Kurahashi, T., Mitsuhashi, W., Inoue, Y., Toyomasu, T., 2008. Germination of photoblastic lettuce seeds is regulated via the control of endogenous physiologically active gibberellin content, rather than of gibberellin responsiveness. J Exp Bot 59, 3383-3393. https://doi.org/10.1093/jxb/ern192.
Schalk, M., Pastore, L., Mirata, M. A., Khim, S., Schouwey, M., Deguerry, F., Pineda, V., Rocci, L., Daviet, L., 2012. Towards a Biosynthetic Route to Sclareol and Amber Odorants. J. Am. Chem. Soc. 134, 18900-18903. https://doi.org/10.1021/ja307404u.
Schepmann, H. G., Pang, J., Matsuda, S. P., 2001. Cloning and characterization of Ginkgo biloba levopimaradiene synthase which catalyzes the first committed step in ginkgolide biosynthesis. Arch Biochem Biophys 392, 263-269. https://doi.org/10.1006/abbi.2001.2438.
Shahi, A., Yu, H., Mafu, S., 2022. Diterpene Biosynthesis in Rice Blast Fungus Magnaporthe. Front Fungal Biol 3, 869823. https://doi.org/10.3389/ffunb.2022.869823.
Shimada, T., Minato, S., Hasegawa, Y., Miyamoto, K., Minato, Y., Shenton, M. R., Okada, K., Kawaide, H., Toyomasu, T., 2023. Characterization of diterpene synthase genes in Brachypodium distachyon, a monocotyledonous model plant, provides evolutionary insight into their multiple homologs in cereals. Biosci Biotechnol Biochem 88, 8-15. https://doi.org/10.1093/bbb/zbad146.

Smanski, M. J., Yu, Z., Casper, J., Lin, S., Peterson, R. M., Chen, Y., Wendt-Pienkowski, E., Rajski, S. R., Shen, B., 2011. Dedicated ent-kaurene and ent-atiserene synthases for platensimycin and platencin biosynthesis. Proc Natl Acad Sci U S A 108, 13498-13503. https://doi.org/10.1073/pnas.1106919108.
Smith, M. W., Yamaguchi, S., Ait-Ali, T., Kamiya, Y., 1998. The first step of gibberellin biosynthesis in pumpkin is catalyzed by at least two copalyl diphosphate synthases encoded by differentially regulated genes. Plant Physiol 118, 1411-1419. https://doi.org/10.1104/pp.118.4.1411.
Stowell, E. A., Ehrenberger, M. A., Lin, Y. L., Chang, C. Y., Rudolf, J. D., 2022. Structure-guided product determination of the bacterial type II diterpene synthase Tpn2. Commun Chem 5, 146. https://doi.org/10.1038/s42004-022-00765-6.
Su, P., Guan, H., Zhao, Y., Tong, Y., Xu, M., Zhang, Y., Hu, T., Yang, J., Cheng, Q., Gao, L., Liu, Y., Zhou, J., Peters, R. J., Huang, L., Gao, W., 2018. Identification and functional characterization of diterpene synthases for triptolide biosynthesis from Tripterygium wilfordii. Plant J 93, 50-65. https://doi.org/10.1111/tpj.13756.
Su, P., Tong, Y., Cheng, Q., Hu, Y., Zhang, M., Yang, J., Teng, Z., Gao, W., Huang, L., 2016. Functional characterization of ent-copalyl diphosphate synthase, kaurene synthase and kaurene oxidase in the Salvia miltiorrhiza gibberellin biosynthetic pathway. Sci Rep 6, 23057. https://doi.org/10.1038/srep23057.
Sugai, Y., Ueno, Y., Hayashi, K., Oogami, S., Toyomasu, T., Matsumoto, S., Natsume, M., Nozaki, H., Kawaide, H., 2011. Enzymatic (13)C labeling and multidimensional NMR analysis of miltiradiene synthesized by bifunctional diterpene cyclase in *Selaginella moellendorffii*. J Biol Chem 286, 42840-42847. https://doi.org/10.1074/jbc.M111.302703.
Sun, T. P., Kamiya, Y., 1994. The Arabidopsis GA1 locus encodes the cyclase *ent*-kaurene synthetase A of gibberellin biosynthesis. Plant Cell 6, 1509-1518. https://doi.org/10.1105/tpc.6.10.1509.
Sun, W., Leng, L., Yin, Q., Xu, M., Huang, M., Xu, Z., Zhang, Y., Yao, H., Wang, C., Xiong, C., Chen, S., Jiang, C., Xie, N., Zheng, X., Wang, Y., Song, C., Peters, R. J., Chen, S., 2019. The genome of the medicinal plant Andrographis paniculata provides insight into the biosynthesis of the bioactive diterpenoid neoandrographolide. Plant J 97, 841-857. https://doi.org/10.1111/tpj.14162.
Tasnim, S., Gries, R., Mattsson, J., 2020. Identification of Three Monofunctional Diterpene Synthases with Specific Enzyme Activities Expressed during Heartwood Formation in Western Redcedar (Thuja plicata) Trees. Plants (Basel) 9 https://doi.org/10.3390/plants9081018.
Toyomasu, T., Kagahara, T., Hirose, Y., Usui, M., Abe, S., Okada, K., Koga, J., Mitsuhashi, W., Yamane, H., 2009. Cloning and characterization of cDNAs encoding ent-copalyl diphosphate synthases in wheat: insight into the evolution of rice phytoalexin biosynthetic genes. Biosci Biotechnol Biochem 73, 772-775. https://doi.org/10.1271/bbb.80781.
Toyomasu, T., Niida, R., Kenmoku, H., Kanno, Y., Miura, S., Nakano, C., Shiono, Y., Mitsuhashi, W., Toshima, H., Oikawa, H., Hoshino, T., Dairi, T., Kato, N., Sassa, T., 2008. Identification of diterpene

biosynthetic gene clusters and functional analysis of labdane-related diterpene cyclases in Phomopsis amygdali. Biosci Biotechnol Biochem 72, 1038-1047. https://doi.org/JST.JSTAGE/bbb/70790 [pii].
Tsavkelova, E. A., Bomke, C., Netrusov, A. I., Weiner, J., Tudzynski, B., 2008. Production of gibberellic acids by an orchid-associated Fusarium proliferatum strain. Fungal Genet Biol 45, 1393-1403. https://doi.org/10.1016/j.fgb.2008.07.011.
Tudzynski, B., Kawaide, H., Kamiya, Y., 1998. Gibberellin biosynthesis in Gibberella fujikuroi: cloning and characterization of the copalyl diphosphate synthase gene. Curr. Gent. 34, 234-240. https://doi.org/10.1007/s002940050392.
Vogel, B. S., Wildung, M. R., Vogel, G., Croteau, R., 1996. Abietadiene synthase from grand fir (Abies grandis). cDNA isolation, characterization, and bacterial expression of a bifunctional diterpene cyclase involved in resin acid biosynthesis. J Biol Chem 271, 23262-23268. https://doi.org/10.1074/jbc.271.38.23262.
Wang, L., Chun, M., Mingcai, Z., Mingwei, D., Xiaoli, T., Li, Z., 2014. Effect of Silencing *Gh*CPS on the Growth and Endogenous Hormone Content of Cotton Seedlings (*Gossypium hirsutum* L.). Cotton Science 26, 189-196. https://doi.org/10.11963/cs140301.
Wu, T. J., Lin, C. C., Ma, L. T., Yang, C. K., Ho, C. L., Wang, S. Y., Chu, F. H., 2024. Functional identification of specialized diterpene synthases from *Chamaecyparis obtusa* and *C. obtusa* var. *formosana* to illustrate the putative evolution of diterpene synthases in Cupressaceae. Plant Sci 344, 112080. https://doi.org/10.1016/j.plantsci.2024.112080.
Wu, Y., Zhou, K., Toyomasu, T., Sugawara, C., Oku, M., Abe, S., Usui, M., Mitsuhashi, W., Chono, M., Chandler, P. M., Peters, R. J., 2012. Functional characterization of wheat copalyl diphosphate synthases sheds light on the early evolution of labdane-related diterpenoid metabolism in the cereals. Phytochemistry 84, 40-46. https://doi.org/10.1016/j.phytochem.2012.08.022.
Xie, P., Ma, M., Rateb, M. E., Shaaban, K. A., Yu, Z., Huang, S. X., Zhao, L. X., Zhu, X., Yan, Y., Peterson, R. M., Lohman, J. R., Yang, D., Yin, M., Rudolf, J. D., Jiang, Y., Duan, Y., Shen, B., 2014. Biosynthetic potential-based strain prioritization for natural product discovery: a showcase for diterpenoid-producing actinomycetes. Journal of natural products 77, 377-387. https://doi.org/10.1021/np401063s.
Xu, M., Hillwig, M. L., Lane, A. L., Tiernan, M. S., Moore, B. S., Peters, R. J., 2014. Characterization of an orphan diterpenoid biosynthetic operon from Salinispora arenicola. Journal of natural products 77, 2144-2147. https://doi.org/10.1021/np500422d.
Xu, M., Hillwig, M. L., Prisic, S., Coates, R. M., Peters, R. J., 2004. Functional identification of rice *syn*-copalyl diphosphate synthase and its role in initiating biosynthesis of diterpenoid phytoalexin/allelopathic natural products. Plant J. 39, 309-318. https://doi.org/10.1111/j.1365-313X.2004.02137.x.

Xu, M., Hillwig, M. L., Tiernan, M. S., Peters, R. J., 2017. Probing Labdane-Related Diterpenoid Biosynthesis in the Fungal Genus Aspergillus. Journal of natural products 80, 328-333. https://doi.org/10.1021/acs.jnatprod.6b00764.
Xu, M., Jia, M., Hong, Y. J., Yin, X., Tantillo, D. J., Proteau, P. J., Peters, R. J., 2018. Premutilin Synthase: Ring Rearrangement by a Class II Diterpene Cyclase. Org Lett 20, 1200-1202. https://doi.org/10.1021/acs.orglett.8b00121.
Yamada, Y., Komatsu, M., Ikeda, H., 2016. Chemical diversity of labdane-type bicyclic diterpene biosynthesis in Actinomycetales microorganisms. The Journal of antibiotics 69, 515-523. https://doi.org/10.1038/ja.2015.147.
Yamane, M., Minami, A., Liu, C., Ozaki, T., Takeuchi, I., Tsukagoshi, T., Tokiwano, T., Gomi, K., Oikawa, H., 2017. Biosynthetic Machinery of Diterpene Pleuromutilin Isolated from Basidiomycete Fungi. Chembiochem 18, 2317-2322. https://doi.org/10.1002/cbic.201700434.
Yang, R., Du, Z., Qiu, T., Sun, J., Shen, Y., Huang, L., 2021. Discovery and Functional Characterization of a Diverse Diterpene Synthase Family in the Medicinal Herb Isodon lophanthoides Var. gerardiana. Plant & cell physiology 62, 1423-1435. https://doi.org/10.1093/pcp/pcab089.
Yu, J., Shiraishi, T., Taizoumbe, K. A., Karasuno, Y., Yoshida, A., Nishiyama, M., Dickschat, J. S., Kuzuyama, T., 2025. Mechanistic Characterization of Diterpene Synthase Pairs for Tricyclic Diterpenes from Cyanobacteria. J Am Chem Soc 147, 11896-11905. https://doi.org/10.1021/jacs.4c16710.
Zerbe, P., Chiang, A., Dullat, H., O'Neil-Johnson, M., Starks, C., Hamberger, B., Bohlmann, J., 2014. Diterpene synthases of the biosynthetic system of medicinally active diterpenoids in Marrubium vulgare. Plant J 79, 914-927. https://doi.org/10.1111/tpj.12589.
Zerbe, P., Chiang, A., Yuen, M., Hamberger, B., Draper, J. A., Britton, R., Bohlmann, J., 2012. Bifunctional *cis*-abienol synthase from *Abies balsamea* discovered by transcriptome sequencing and its implications for diterpenoid fragrance production. J. Biol. Chem. 287, 12121-12131. https://doi.org/10.1074/jbc.M111.317669.
Zerbe, P., Hamberger, B., Yuen, M. M., Chiang, A., Sandhu, H. K., Madilao, L. L., Nguyen, A., Hamberger, B., Bach, S. S., Bohlmann, J., 2013. Gene discovery of modular diterpene metabolism in nonmodel systems. Plant Physiol 162, 1073-1091. https://doi.org/10.1104/pp.113.218347.
Zerbe, P., Rodriguez, S. M., Mafu, S., Chiang, A., Sandhu, H. K., O'Neil-Johnson, M., Starks, C. M., Bohlmann, J., 2015. Exploring diterpene metabolism in non-model species: transcriptome-enabled discovery and functional characterization of labda-7,13E-dienyl diphosphate synthase from Grindelia robusta. Plant J 83, 783-793. https://doi.org/10.1111/tpj.12925.
